# Mechanism of Methyl Transfer Reaction between CH_3_Co(dmgBF_2_)_2_py and PPh_3_Ni(Triphos)

**DOI:** 10.3390/molecules29143335

**Published:** 2024-07-16

**Authors:** Patrycja Sitek, Piotr Lodowski, Maria Jaworska

**Affiliations:** Institute of Chemistry, University of Silesia in Katowice, Szkolna 9, 40-006 Katowice, Polandpiotr.lodowski@us.edu.pl (P.L.)

**Keywords:** cobalt complex, nickel complex, methyl transfer, DFT, dispersion correction

## Abstract

DFT calculations were performed for the methyl group transfer reaction between CH_3_Co (dmgBF_2_)py and PPh_3_Ni(Triphos). The reaction mechanism and its energetics were investigated. This reaction is relevant to the catalytic mechanism of the enzyme acetyl coenzyme A synthase. BP86 and PBE functionals and dispersion corrections were used. It was found that intermolecular interactions are very important for this reaction. The influence of the solvent on the reaction was studied.

## 1. Introduction

Cobalamin- and cobinamide-dependent methyltransferases are enzymes which use methylcobalamin as a methylating factor [1,2,3]. Examples of such enzymes are methionine synthase, methanol-coenzyme M methyltransferase, and corrinoid iron-sulfur protein (CoFeSP) from acetyl coenzyme A synthase (ACS). CoFeSP is an enzyme which transfers a methyl group from the cobalt atom in methylcobinamide to the nickel atom in the ACS enzyme. Methylcobalamin occurs in CoFeSP in the base-off form (cobinamide), where the axial benzimidazole is replaced by a water ligand [4,5].

This unique methyl transfer reaction, where metals act as donors and acceptors of the methyl group, is found in the Ljungdahl–Wood pathway of autotrophic carbon fixation in various bacteria and archaea [6]. Acetyl-CoA is synthesized at the Ni-Ni-[4Fe-4S] cluster (the A-cluster) of acetyl-CoA synthase (ACS) through condensation of coenzyme-A (CoASH) with CO and the methyl group from CH_3_-Cob(III)alamin of the corrinoid-iron-sulfur protein (CoFeSP) [7,8]. A key step of such synthesis is the transfer of the methyl group from CoFeSP to the proximal Ni atom in the active site of ACS [9]. This reaction proceeds according to the following equation:

(1) $$CH_{3}-Co\left(III\right)FeSP+CO+CoASH⇌CH_{3}CO-SCoA+Co\left(I\right)FeSP+H^{+}$$

The occurrence of Ni(0) [10,11] or Ni(I) [12] in reaction (1) of ACS was postulated. Since the mechanism of catalytic action of the ACS enzyme is not fully understood [10,13,14,15], models of methylation reactions involving nickel complexes and various methylation factors are being examined experimentally [11,16,17,18,19,20,21,22]. Likewise, many complexes relevant to ACS enzyme are investigated experimentally [23,24,25,26,27] and computationally [28,29,30,31]. One example of methylation reactions with nickel participation are [12,31]:

(2) $$\begin{array}{cc} \; & Ni\left(Triphos\right)PPh_{3}+CH_{3}Co{\left(dmgBF_{2}\right)}_{2}py+sol⟶\\ \; & CH_{3}Ni{\left(Triphos\right)}^{+}+Co{\left(dmgBF_{2}\right)}_{2}sol^{-}+PPh_{3}+py, \end{array}$$

where Triphos stands for bis(diphenylphosphinoethyl)phenylphosphine ligand, dmg denotes dimethylglyoxime, and py is pyridine. In reaction (2), nickel in Ni(Triphos)PPh_3_ is in the Ni(0) oxidation state. In the course of the reaction, the py ligand is exchanged for the solvent molecule.

In general, two mechanisms, S_N_2 and radical, are possible in methyl transfer reactions with cobalamin participation [31,32,33]. Reaction (2) involves methylation of nickel(0) complexes. For the radical mechanism, in the first step, the methyl derivative should be reduced by the methyl acceptor; thus, the homolytic cleavage of the Co-CH_3_ bond is initiated by electron transfer between reactants. The radical mechanism is therefore possible when the methyl acceptor is able to reduce the methyl donor. Reaction 2 is an example of a nickel complex in the zero oxidation state. The oxidation state of nickel in methylation in acetyl coenzyme A synthase remains controversial. Proposed mechanisms involve Ni(0) and Ni(1). Reaction 2 proves that Ni(0) methylation is possible in model systems and that makes it probable that such a mechanism could also occur in a biological system and thus makes this reaction interesting. Our goal was to investigate the mechanism and energetics of this reaction with the DFT method.

The properties of the cobalt-nickel complexes studied in this work were previously investigated by DFT [34]. The mechanism of action of acetyl coenaym A synthase was studied computationally using the DFT method [35,36,37,38,39].

## 2. Computational Method

The calculations were carried out with the use of Gaussian16 [40] and ORCA 5.0.4 [41,42,43] programs. The DFT method was used in the calculations with BP86 [44,45] and PBE [46] functionals together with the def2-TZVP basis set for metal atoms and TZVP basis for the remaining atoms [47]. For theoretical modeling of reactions involving transition metal atoms with the use of the DFT method, it is very important to apply a functional which properly describes the electronic structure of the reactants, giving results comparable with experiment. This is especially important for methyl-metal binding energy and oxidation–reduction properties of the reacting complexes. It was shown that nonhybrid functionals allow us to obtain a good description of the cobalt-methyl bond in alkylcobalamins, while the hybrid functionals significantly underestimate the energy of this bond [48,49,50]. The nonhybrid functionals also give good estimation for redox potentials for transition metal complexes [51,52,53]. In calculations, the effect of the environment was taken into account by the PCM solvent model [54,55,56], with acetonitrile (ϵ = 35.688) as the solvent. Acetonitrile was used because it is the solvent in reaction 2. Several dispersion corrections were applied, including G3BJ [57,58], G4 [59], and nonlocal self-consistent functional VV10 (SCNL) [60,61]. The atomic charges were calculated according to NBO population analysis [62].

**Figure 1:**
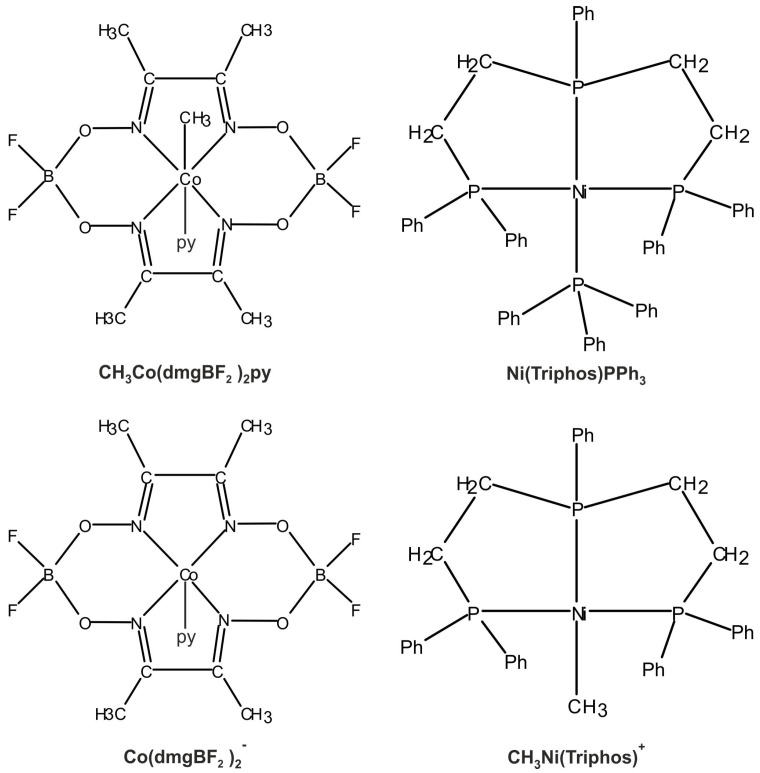
Lewis structures of the basic structural elements of molecules important in the reaction (2).

**Figure 2:**
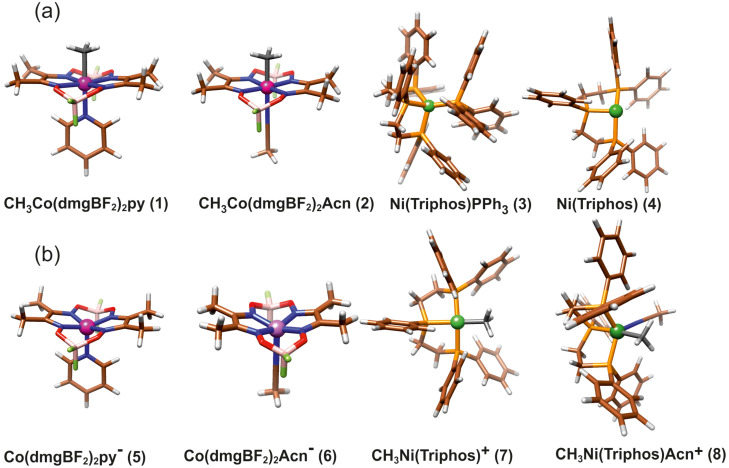
The structure of molecules considered in study: (**a**) Reagents, (**b**) Products.

The main molecular structures involved in the mechanism of reaction under study are presented in Figure 1 in the form of the Lewis structures, and their 3D spatial forms are shown in Figure 2. In the case of the nickel complex, various possible structures differing in the number of ligands as well as their form were considered.

The geometries of all species were fully optimized without any constraints of geometric parameters. For optimized geometries, using the harmonic approximation the calculations of normal mode frequencies was performed in order to determine the thermodynamic corrections and establish that the obtained optimal geometric structures correspond to the stationary points on the potential energy surface (PES). Based on the results of the vibrational analysis, it was found that the all optimized structures correspond to the local minimum on the PES. Since the thermodynamic corrections are determined basing on the formalism that takes into account the state of the system in the gas phase, many different corrections were applied in the literature for obtaining more realistic values of the rotational and translational entropy in the solution [63,64,65,66,67,68,69,70,71,72]. We used the method in which the translational and rotational entropy contributions to Gibbs energy were scaled down to 50% of their gas phase values.

The potential energy curves that described the energetics of the reaction path were determined as functions of the nickel-methyl distance. This distance was frozen at a specific value while the remaining geometry parameters were optimized.

## 3. Results

Two approaches were used in the calcultions, that is, BP86 without dispersion corrections and the PBE functional with dispersion corrections. The total energies for all molecules and methods used in this work are collected in Table S1 in Supporting Materials.

### 3.1. BP86 Calculations without Dispersion

#### 3.1.1. Geometry of Species and Energetics of Reaction Path

**Figure 3:**
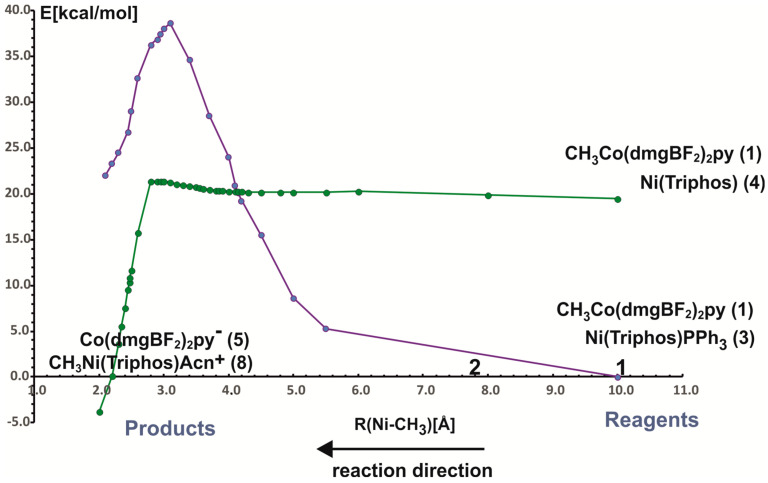
The BP86 calculated energy curves as a function of Ni-CH_3_ distance. Numbering of molecules refers to Figure 2. The green curve represents Ni(triphos) without PPh_3_ and CH_3_Co(dmgBF_2_)_2_py; the purple one represents the version with PPh_3_ ligand coordinated to nickel (complex 3 in Figure 2).

In the reaction of methyl transfer, the PPh_3_ ligand connected to nickel undergoes dissociation during methyl exchange between cobalt and nickel. Simultaneously, it is possible to take into account that the triphenylphosphine ligand can be exchanged for a solvent molecule, and we assume that the end product of the reaction, which is a methyl nickel complex, is a five-coordinate complex containing a solvent molecule (ACN) as a ligand. Taking into account that nickel atoms are coordinated by different numbers and types of ligands, we performed calculations for two energy curves with different ligands attached to nickel. In these calculations, the nickel-methyl carbon distance was kept constant at a given value in the range between 10 Å and 2 Å, and the remaining parameters were optimized. The obtained potential energy curves as a function of Ni-CH_3_ distance are depicted in Figure 3.

The two potential energy curves in Figure 3 correspond to different nickel coordination at the reagent side. The green PEC applies to the situation where the nickel complex as a reagent is a three-coordinated complex without PPH_3_ ligand, whereas the second PEC corresponds to a reagent structure in which triphenylphosphine is attached to nickel. The crossing of both curves represents the geometry where phosphine departs from nickel and is being replaced by the methyl group. It occurs quite early on the reaction path at a Ni-methyl distance larger than 4 Å. The energy barrier to the point where the curves intersect is about 20 kcal/mol and it comes mainly from the phosphine ligand dissociation. After PEC crossing, between 4.1 Å and 2.8 Å there is a plateau on a curve with lower energy. At a Ni-CH_3_ distance of about 2.8 Å the energy lowers very quickly, which is associated with forming of the Ni-CH_3_ bond. At a distance of 2.0 Å, an ACN ligand was attached to the Ni product and the geometry of the complex was re-optimized. The reaction is exothermic only after the acetonitrile molecule is coordinated with nickel to give CH_3_Ni(Triphos)Acn^+^ complex. The reaction energy is equal to 3.8 kcal/mol. The detailed analysis of reaction energetics is carried out in Section 4.

#### 3.1.2. Charges

**Figure 4:**
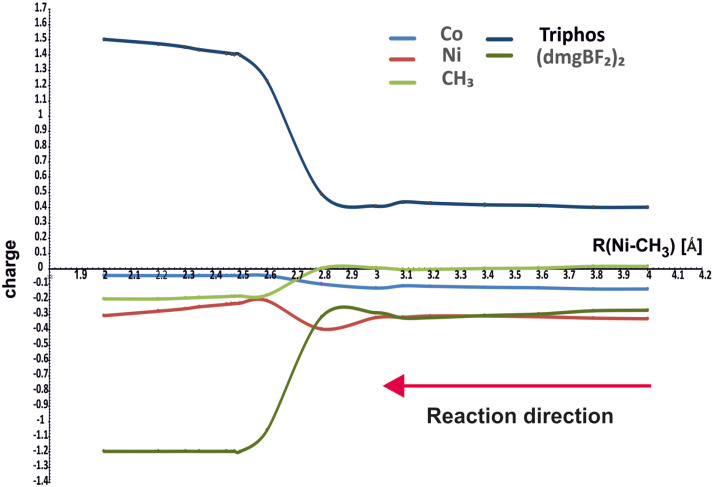
NBO charges on different parts of the molecules as functions of Ni-CH_3_ distance.

Figure 4 shows how the NBO charges on different molecular fragments change along the reaction path. The charges are drawn for the minimum energy curves in Figure 3, that is, for the purple curve above 4.1 Å and for the green one for the Ni-CH_3_ distances smaller than 4.1 Å.

It can be seen that the charges on methyl and cobalt do not change much during the course of the reaction; methyl acquires the charge about −0.2 e after bonding with nickel. The nickel ion shows a substantially negative charge, about −0.8 e, in the starting complex with PPh_3_, reaching about −0.3 e in the complex with the methyl group. However, the largest changes occur on the (dmgBF_2_)_2_ (from small negative to about −1.2 e) and Triphos (from small positive to about 1.2 e) ligands. Hence, the changes in charges do not confirm that methyl is transferred as a positive group, but it retains a generally small charge, even finally negative. The changes involve rather the flow of electron density between coligands through the metal ions.

### 3.2. DFT with Dispersion Corrections

We studied reaction (2) with BP86/D3BJ, PBE/D3BJ, PBE/D4, and PBE/SCNL combinations of functionals and dispersion corrections. In all cases, the reagents and products were optimized with the use of the specific method. The energy of all reactions that are considered further is defined as

(3) ΔE=E(Reagents)−E(Products).

To analyze the energetics of reaction (2), we calculated the energy of four possible reaction mechanisms (4)–(7):

(4) $$\begin{array}{cc} & Ni\left(Triphos\right)\left(PPh_{3}\right)+CH_{3}Co{\left(dmgBF_{2}\right)}_{2}py\to CH_{3}Ni{\left(Triphos\right)}^{+}+Co{\left(dmgBF_{2}\right)}_{2}py^{-}+PPh_{3} \end{array}$$

(5) $$\begin{array}{cc} & Ni\left(Triphos\right)\left(PPh_{3}\right)+CH_{3}Co{\left(dmgBF_{2}\right)}_{2}py\to CH_{3}Ni{\left(Triphos\right)}^{+}\cdots Co{\left(dmgBF_{2}\right)}_{2}py^{-}+PPh_{3} \end{array}$$

(6) $$\begin{array}{cc} & Ni\left(Triphos\right)\left(PPh_{3}\right)+CH_{3}Co{\left(dmgBF_{2}\right)}_{2}py+Acn\to CH_{3}Ni\left(Triphos\right)Acn^{+}+Co{\left(dmgBF_{2}\right)}_{2}py^{-}+PPh_{3} \end{array}$$

(7) $$\begin{array}{cc} & Ni\left(Triphos\right)\left(PPh_{3}\right)+CH_{3}Co{\left(dmgBF_{2}\right)}_{2}py+Acn\to CH_{3}Ni\left(Triphos\right)Acn^{+}\cdots Co{\left(dmgBF_{2}\right)}_{2}py^{-}+PPh_{3} \end{array}$$

**Figure 5:**
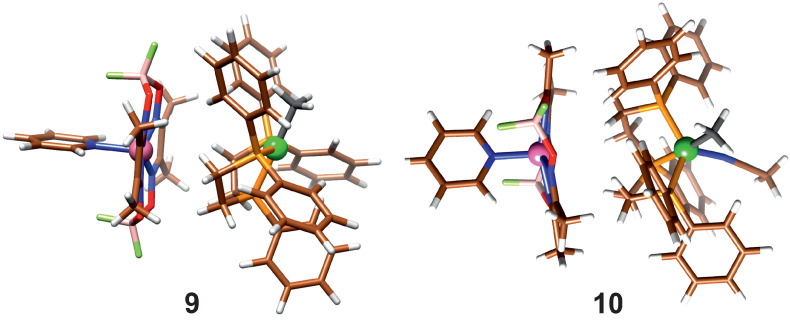
Association of product molecules with (10) and without (9) coordinated Acn.

In reactions (5) and (7), the product association is taken into account; they differ in whether a solvent molecule is attached. When the dispersion correction is considered, the product molecules form an association complex, shown in Figure 5.

The calculated reaction energy values are gathered in Table 1.

Reactions (4) and (6) are exothermic only for the BP86 method. The energy effect of these reactions changes to strongly endothermic when dispersion corrections are included in the calculations. Considering the simplest mechanism, taking into account only the transfer of the methyl group and the dissociation of the PPh3 ligand from the nickel complex (reaction (4)), the calculated energy and Gibbs free energy of this reaction are −31 and −23 kcal/mol, respectively. When it is assumed that in the reaction environment, the acetonitrile molecule present in excess can be an additional ligand in the methylated nickel complex (reaction (6)), the calculated ΔE and ΔG values are −18 and −15 kcal/mol, respectively. Thus, the solvent molecule, as a ligand, can have some stablilizing effect on the energetics of the reaction, although the effect is not sufficient to achieve an exothermic energy balance of the reaction. Also, presuming that the reaction products form associations (reaction (5)), a reduction in the negative value of ΔE and ΔG can be observed in the computational results compared to the mechanism that does not take into account the association. Product association thus also has a stabilizing effect on the energetics of the reaction. When both effects are taken into account, i.e., the product molecule association and the coordination of acetonitrile to CH_3_Ni(Triphos), the reaction energy ΔE and the free energy ΔG become slightly positive values and are 1.1 and 1.85 kcal/mol. Similar characteristics of reaction energetics have been obtained using the PBE functional and different variants of dispersion corrections in the calculations (Table S2 in Supporting Materials). The obtained results vary between −1.1 kcal/mol (BP86/D3BJ) and 2.9 kcal/mol (PBE/SCNL). Consequently, according to the calculation results, the reaction is practically thermoneutral; however, it is known from experiment that reaction (2) is very fast.

**Figure 6:**
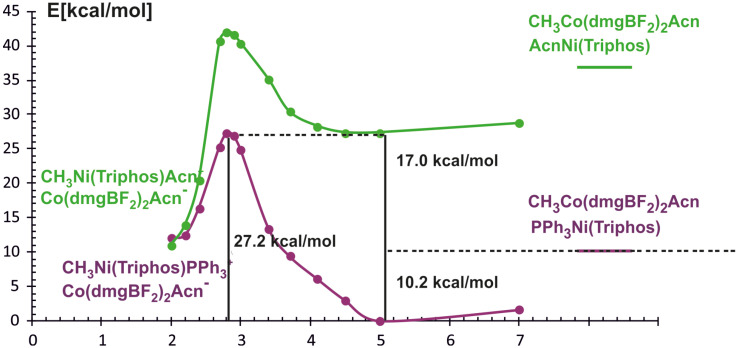
Energy curves for methyl transfer reaction calculated with PBE/D3BJ method as a function of Ni-CH_3_ distance. The purple curve refers to Ni(Triphos)PPh_3_ as reagent, the green one to Ni(Triphos)Acn.

By analogy with the BP86 functional calculations, the energy profile of the considered reaction was determined as a function of the Ni-methyl distance using the PBE gradient functional and the D3BJ dispersion correction. With respect to the reactants occurring in the associate form, the calculated energy profile of the methyl transfer reaction between Co and Ni complexes is shown in Figure 6.

A characteristic feature of the obtained PECs is the occurrence of minima when reagents approach each other, which is a result of the inclusion of dispersion interactions. In addition, it can be noticed that the two curves cross at about 2.2 Å which corresponds to PPh_3_ dissociation and Acn coordination. This is in contrast with the BP86 results where phosphine departs at ∼4.1 Å. The inclusion of dispersive interactions in the calculations, as well as the fact that the reactants can occur in the form of associates, consequently changes the energy characteristics of the reaction path; nevertheless, the total energy effect of the process remains almost thermodynamically neutral.

### 3.3. Solvation of Reagents and Products

**Figure 7:**
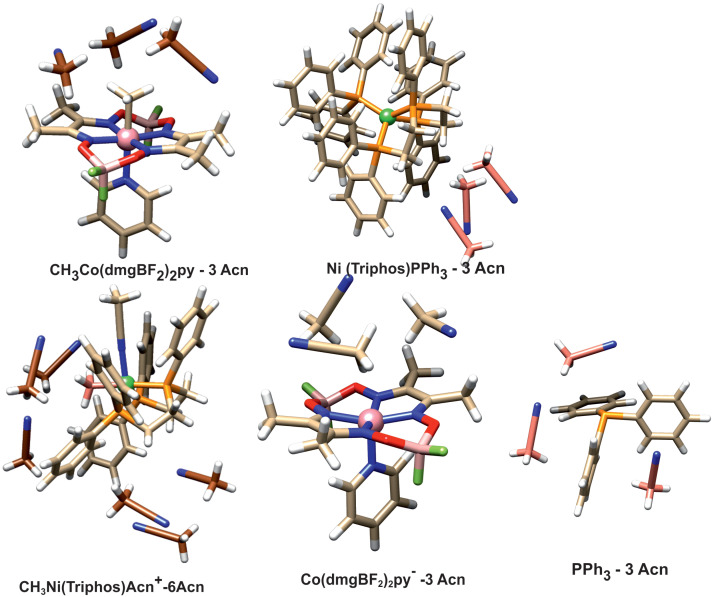
Structure of the solvated complexes and PPh_3_ ligand.

The question arises whether the product association persists in solution. To investigate this, the energy associated with the solvation of reactant and product molecules was determined. To simulate the interaction with the solvent, structural models were used in which the direct presence of several acetonitrile molecules as a solvent was taken into account. Solvation was investigated with PBE/D3BJ calculations, and in these calculations, a continuous model of the solvent was also used. The geometries of the solvated species are presented in Figure 7.

The acetonitrile molecules were added to these parts of structure surfaces, which were engaged in forming associated complexes or intermolecular interactions. In the case of the CH_3_Ni(Triphos)Acn^+^ complex, there are two such sites. One is the area around the nickel-bound phosphine ligand, and the other is the area on the side of the methyl ligand. The latter area corresponds to the space between the interacting nickel and cobalt complexes occurring in the form of an associated structure. Thus, in the case of the CH_3_Ni(Triphos)Acn^+^ complex, six molecules of solvent were used in the calculation. The calculated solvation energies are gathered in Table 2.

The estimated stabilization energy is equal to 27.3 kcal/mol. However, the quantity calculated in this way depends on the number of solvent molecules taken into account in the calculations. On the other hand, it can be concluded how much influence solvation has on the energy of the studied reaction.

## 4. Discussion

### Analysis of Energy of the Methyl Transfer Reaction

To check the origins of the energy differences in used functionals and dispersion methods, the energies of reaction (6) have been described as the sum of the energies of elementary reactions depicted in Equations (8)–(16).

(8) $$\begin{array}{cccc} & Ni\left(Triphos\right)PPh_{3}\to Ni\left(Triphos\right)+PPh_{3}\; & & E_{B}\left(Ni\left(Triphos\right)-PPh_{3}\right) \end{array}$$

(9) $$\begin{array}{cccc} & Ni\left(Triphos\right)PPh_{3}^{+•}\to Ni{\left(Triphos\right)}^{+•}+PPh_{3}\; & & E_{B}\left(Ni{\left(Triphos\right)}^{+•}-PPh_{3}\right) \end{array}$$

(10) $$\begin{array}{cccc} & CH_{3}Co{\left(dmgBF_{2}\right)}_{2}py^{-•}\to CH_{3}^{•}+Co{\left(dmgBF_{2}\right)}_{2}py^{-}\; & & E_{B}\left(Co{\left(dmgBF_{2}\right)}_{2}py^{-}-CH_{3}\right) \end{array}$$

(11) $$\begin{array}{cccc} & CH_{3}Co{\left(dmgBF_{2}\right)}_{2}py\to CH_{3}^{•}+Co{\left(dmgBF_{2}\right)}_{2}py^{•}\; & & E_{B}\left(Co{\left(dmgBF_{2}\right)}_{2}py^{•}-CH_{3}\right) \end{array}$$

(12) $$\begin{array}{cccc} & CH_{3}Ni{\left(Triphos\right)}^{+}\to CH_{3}^{•}+Ni{\left(Triphos\right)}^{+•}\; & & E_{B}\left(Ni{\left(Triphos\right)}^{+•}-CH_{3}\right) \end{array}$$

(13) $$\begin{array}{cccc} & CH_{3}Ni\left(Triphos\right)Acn^{+}\to CH_{3}Ni{\left(Triphos\right)}^{+}+Acn\; & & E_{B}\left(CH_{3}Ni{\left(Triphos\right)}^{+}-Acn\right) \end{array}$$

(14) $$\begin{array}{cccc} & CH_{3}Co{\left(dmgBF_{2}\right)}_{2}py+Acn\to CH_{3}Co{\left(dmgBF_{2}\right)}_{2}Acn+py\; & & E_{B}\left(CH_{3}Co{\left(dmgBF_{2}\right)}_{2}Acn-py\right) \end{array}$$

(15) $$\begin{array}{cccc} & Ni\left(Triphos\right)PPh_{3}\to Ni\left(Triphos\right)PPh_{3}^{+•}+e\; & & E^{0}\left(Ni\left(Triphos\right)PPh_{3}^{+•}/Ni\left(Triphos\right)PPh_{3}\right) \end{array}$$

(16) $$\begin{array}{cccc} & CH_{3}Co{\left(dmgBF_{2}\right)}_{2}py+e\to CH_{3}Co{\left(dmgBF_{2}\right)}_{2}py^{-•}\; & & E^{0}\left(CH_{3}Co{\left(dmgBF_{2}\right)}_{2}py/CH_{3}Co{\left(dmgBF_{2}\right)}_{2}py^{-•}\right) \end{array}$$

The calculated energies and free energies of the above partial reactions are gathered in Table 3.

Based on the Hess’s law, the energetics effect of reaction (6) can be expressed as follows:

(17) $$\begin{array}{c} E(6)=(E(16)-E(15))+(E(12)-E(10))+(E(13)-E(9)) \end{array}$$

In turn, in the energies of reactions (6) and (7), the energetic cost of pyridyne and Acn ligand exchange in the cobalt complex is taken into account:

(18) $$\begin{array}{cc} \; & E(18)=E(6)-E(14) \end{array}$$

(19) $$\begin{array}{cc} \; & E(19)=E(7)-E(14) \end{array}$$

where E(14) denotes the difference in pyridine and Acn binding energy. It can be seen that ligand exchange on the cobalt complex Table 3, reactions (18) and (19)) shifts the energy balance towards more endothermic values in relation to the energetics of reactions (6) and (7), presented in Table 1. This change applies to both the energy and free energy of these reactions. The bonding of cobalt with acetonitrile is weaker than with pyridine; however, this ligand exchange takes place because of large acetonitrile concentration, considering that it is a solvent in this reaction. Once this ligand exchange occurs, it is irreversible.

The E(6) energy values for different methods used in calculations are showed in Table 4. In general, except for the result obtained with the BP86 function, the calculated energy effect of reaction (6) is negative in the range of −0.4 to −25 kcal/mol. The closest result of ΔE = −0.4 kcal/mol to the ΔE value, obtained in calculations with BP86, corresponds to calculations using the PBE functional. In other cases, when correction for dispersion interactions was applied, the results indicated a significantly endothermic character of the reaction. On the other hand, dispersive interactions here have a significant impact on the stabilization of the associate structures of the complexes. As presented in Section 3.2, direct consideration of the association gives a result corresponding to the weak exothermicity of the methyl group transfer process between the considered cobalt and nickel complexes (Table 1, reaction (7)).

**Figure 8:**
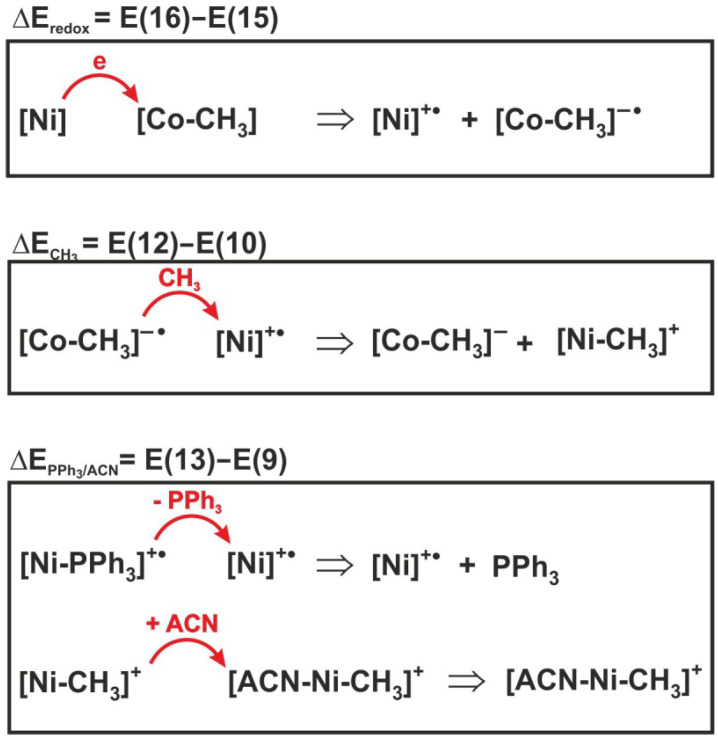
Reactions whose energies are shown in Table 4.

To determine the contribution of individual elementary reactions to the total energetics of reaction (6), the energy effect of the three individual processes included in expression (17) was analyzed. For illustrative purposes, the sense of individual energy differences is shown in Figure 8. Thus, E(16)–E(15) is the difference in redox potentials of the complexes, denoted as $\Delta E_{redox}$. This is the energy effect associated with electron transfer between the nickel and cobalt complexes. The $\Delta E_{CH_{3}}$ = E(12) − E(10) corresponds to the energy effect of the methyl group transfer, while $\Delta E_{PPh_{3}/ACN}$ is the difference E(13)–E(9) between the dissociation energy of the PPh_3_ group from the reactant and the attachment energy of acetonitrile to the product, i.e., the methylated nickel complex.

The first of the mentioned energy differences, $\Delta E_{redox}$, is completely comparable for the different computational methods used and correlates well with the value derived from experimental data (exp. $\Delta E_{redox}$ = 1 eV). The energetics of the methyl group transfer process, $\Delta E_{CH_{3}}$, also remain largely similar for the various calculation methods and are in the range of 28 to 32 kcal/mol. These are positive values, so for all the methods used, the methyl transfer is an exothermic reaction. Thus, this result confirms that for the studied reaction, the key stage is an energetically preferred process. Also, from the perspective of the free energy, this fact is fully confirmed (Table 4, PBE/D3BJ calculations). In this case, the values of ΔE and ΔG are close to each other and are 30.0 kcal/mol and 28.4 kcal/mol, respectively. On the contrary, the dissociation of the PPh_3_ ligand from the nickel complex in combination with the attachment of the ACN molecule to the product is a fully exothermic process in terms of considerations based on elementary reactions. All $\Delta E_{PPh_{3}/ACN}$ values in Table 4 are negative regardless of the method, with or without the inclusion of dispersion corrections in the calculations, which clearly enhances the endothermicity of this reaction step. This fact can be explained by the expanded molecular structure of the PPh_3_ ligand, which means that the dispersion contribution of the PPh_3_ interaction in the complex structure should be significant. The dissociation of this ligand from nickel is thus an energetically unfavorable process. In the overall energy balance of the considered reaction (6), the endothermicity of the disconnection step of the phosphine ligand is therefore not effectively compensated by the exothermic effect of the nickel methylation process and the solvent molecule coordination in the product.

**Figure 9:**
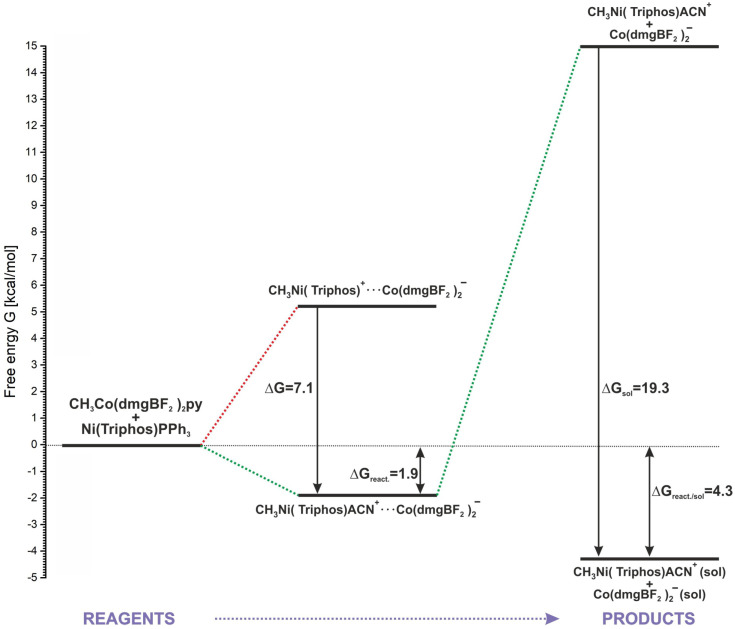
Schematic description of the methyl group transfer reaction.

As it was shown in Section 2, the positive energetic balance of the modeled reaction (2) can be obtained by assuming the association of reactants and products and the attachment of the ACN molecule to the methylation product (Table 1, reaction (7)). Only with this approach are both ΔE and ΔG values slightly positive. However, speaking more generally, this result rather indicates the neutral nature of the reaction energetics. An energy diagram representing the energetics of the key stages of the modeled reaction is shown in Figure 9. The starting point is the separated reactants, whose sum of energy is the baseline (0.0 kcal/mol) in relation to the remaining steps. The association of reactants is undoubtedly an advantageous process, allowing the nickel and cobalt complexes to come closer together. Also, the insertion of a solvent molecule into the coordination sphere of the methylated nickel is favorable in respect of product stabilization. As shown in Figure 9, consideration of the coordination of ACN to nickel lowers the energy of the associated products by about 7 kcal/mol. Associated products form a stable macrostructure, and from the point of view of the reaction energetics, the formation of a conglomerate determines the slight reaction endothermicity. The calculated ΔG of the reaction up to this step is 1.9 kcal/mol. The association energy of the MeNi(triphos)Acn^+^ and Co(dmgBF_2_)_2_py^−^ complexes, estimated from calculations, is about 17 kcal/mol. This is a relatively large value, indicating that a complex conglomerate may exist as a structural form of the resulting products in the reaction. However, it should be noted that the reaction takes place in a solvent environment and the action of solvent molecules can lead to the separation of the associates. As shown by the calculation results presented in Section 3.3, the solvation of separated products is an energetically preferable process, giving a significant contribution to the system stabilization. After taking into account the solvation of the products, the estimated free energy of the process sequence from reactants to substrates is 4.3 kcal/mol, as shown in Figure 9.

## 5. Conclusions

The calculations for the energy of methyl transfer between cobalt and nickel complexes using the DFT method together with dispersion corrections were carried out. On the basis of the performed calculations, the following conclusions can be derived:

- The analysis of reaction energy reveals that acetonitrile solvent participates actively in the course of the reaction by coordination with cobalt and nickel centers.
- The dispersion corrections influence energetics and the mechanism of the reaction by forming the associated product complex and impeding the dissociation of the phospine ligand.
- The methyl group binding energy is larger with nickel than with cobalt, which is in favor of product formation. However, the high binding energy of the phosphine group in the starting nickel complex has an unfavorable impact on the course of the reaction.
- The calculated energy shows that the reaction is basically endothermic. However, the solvation strongly stabilizes the reaction products.

## Figures and Tables

**Table 1:** Total energy (Δ**E**) and total Gibbs free energy (Δ**G**) of the reaction (1)–(4) in kcal/mol.

Reaction	BP86	PBE/D3BJ	PBE/D3BJ
	ΔE	ΔE	ΔG
(4)	0.4	−30.7	−22.88
(5)	–	−12.2	−7.08
(6)	3.8	−17.7	−15.01
(7)	–	1.1	1.85

**Table 2:** Solvation Gibbs free energy for the reagents and products of reaction (2).

		Solvation
		**Energy**
Products	PPh_3_ − 3 Acn	8.40
	Co(dmgBF_2_)_2_py^−^ − 3Acn	9.71
	CH_3_Ni(Triphos)Acn^+^ − 6Acn	19.52
Reagents	CH_3_Co(dmgBF_2_)_2_py ^+^ − 3Acn	6.13
	Ni(Triphos)PPh_3_ − 3Acn	4.21
Solvation		
Stabilization		27.29
energy		

**Table 3:** Reaction energies (Δ**E**) and reaction Gibbs free energies (Δ**G**) in kcal/mol. Entries (15) and (16) are redox potentials in Volts.

	BP86	PBE-D3BJ	
Reaction	ΔE	ΔE	ΔG
	Total reaction energy		
(18)	−1.5	−27.7	−22.94
(19)	–	−8.9	−6.08
	Elementary reaction energy		
(8)	19.4	50.9	42.54
(9)	11.6	44.4	36.82
(10)	19.6	26.6	19.68
(11)	39.3	47.0	36.46
(12)	47.9	56.6	48.07
(13)	3.4	13.0	7.86
(14)	5.3	10.0	7.93
(15) ^(a)^	−0.3	−0.5	−0.42
(16) ^(b)^	−1.0	−1.2	−1.04

^(a)^ Expt −0.1 V (Ref. [31]). ^(b)^ Expt −1.1 V (Ref. [31]).

**Table 4:** Energy of the partial reactions (in kcal/mol).

		E(6)	$\Delta E_{\mathrm{redox}}$	$\Delta E_{{\mathrm{CH}}_{3}}$	$\Delta E_{{\mathrm{PPh}}_{3}/\mathrm{ACN}}$
			eV (kcal/mol)		
BP86	ΔE	3.8	−0.7 (−16.1)	28.3	−8.2
BP86-D3BJ	ΔE	−25.1	−0.59 (−13.6)	32.0	−44.4
PBE	ΔE	−0.4	−0.7 (−16.1)	28.5	−12.4
PBE-D3BJ	ΔE	−17.7	−0.7 (−16.1)	30.0	−31.4
PBE-D3BJ	ΔG	−15.0	−0.62 (−14.3)	28.39	−28.96
PBe-D4	ΔE	−17.6	−0.7 (−16.1)	32.1	−34.1
PBE-SCNL	ΔE	−16.6	−0.7 (−16.1)	31.6	−31.7

## Data Availability

Data are available from the authors upon request.

## Supplementary Materials

The following supporting information can be downloaded at: https://www.mdpi.com/article/10.3390/molecules29143335/s1, Cartesian coordinated of the molecules under study, Table S1: Total energies of molecules considered in the calculations; Table S2: Reaction energies in kcal/mol. Entries a and b are redox potentials in Volts.

## Abbreviations

The following abbreviations are used in this manuscript:

py	pyridine
acn	acetonitrile
dmg	dimethylglioxyme
Triphos	Bis(diphenylphosphinoethyl)phenylphosphine
PEC	Potential Energy Curve
NBO	Natural Bond Orbital
