# Supplementary material for: Mechanism of Methyl Transfer Reaction between CH3Co(dmgBF2)2py and PPh3Ni(Triphos)
Source: Molecules. 2024 Jul 16;29(14):3335. doi: 10.3390/molecules29143335 (PMC11280430; doi:10.3390/molecules29143335)
Supplement: Supplementary file 1 [file molecules-29-03335-s001.zip › molecules-3001670-supplementary.pdf]

# Mechanism of Methyl Transfer Reaction Between $CH_3Co(dmgBF_2)py$ and $PPh_3Ni(triphos)$

**Patrycja Sitek, Piotr Lodowski and Maria Jaworska**

1. Cartesian coordinates of molecules considered in the calculations

**CH3Ni(Triphos)Acn-6Acn**

| | | | |
|---|---|---|---|
| Ni | 2.198297000 | -0.308144000 | 0.483339000 |
| P | 1.494617000 | -2.367999000 | 0.227406000 |
| P | 1.297575000 | -0.399585000 | 2.496228000 |
| P | 0.854116000 | 1.382445000 | 0.115032000 |
| C | -0.647109000 | -2.790146000 | -1.570583000 |
| H | -1.322796000 | -2.523527000 | -0.756304000 |
| C | -1.185232000 | -3.057903000 | -2.832960000 |
| H | -2.265201000 | -2.993622000 | -2.979467000 |
| C | -0.347041000 | -3.391948000 | -3.900879000 |
| H | -0.769293000 | -3.596342000 | -4.886614000 |
| C | 1.035685000 | -3.467142000 | -3.699119000 |
| H | 1.698943000 | -3.726086000 | -4.526947000 |
| C | 1.576876000 | -3.197684000 | -2.440893000 |
| H | 2.655908000 | -3.258867000 | -2.295789000 |
| C | 0.740722000 | -2.861909000 | -1.359738000 |
| C | 3.885777000 | -3.373141000 | 1.265259000 |
| H | 4.065148000 | -2.334744000 | 1.548430000 |
| C | 4.818763000 | -4.364736000 | 1.585050000 |
| H | 5.727849000 | -4.097998000 | 2.127561000 |
| C | 4.593695000 | -5.687712000 | 1.194601000 |
| H | 5.326285000 | -6.461104000 | 1.433323000 |
| C | 3.427953000 | -6.020051000 | 0.492464000 |
| H | 3.248771000 | -7.052889000 | 0.187016000 |
| C | 2.490970000 | -5.032933000 | 0.179755000 |
| H | 1.584860000 | -5.298989000 | -0.367973000 |
| C | 2.717121000 | -3.698598000 | 0.560419000 |
| C | 0.190631000 | -2.698884000 | 1.508714000 |
| H | -0.736146000 | -2.201138000 | 1.189816000 |
| H | -0.006381000 | -3.777591000 | 1.587350000 |
| C | 0.680871000 | -2.108081000 | 2.831194000 |
| H | 1.528212000 | -2.695406000 | 3.214288000 |
| H | -0.105173000 | -2.092864000 | 3.600002000 |
| C | 2.786321000 | 1.426341000 | 3.954425000 |
| H | 2.467176000 | 2.139382000 | 3.190525000 |
| C | 3.657513000 | 1.844880000 | 4.960038000 |
| H | 3.998244000 | 2.881855000 | 4.982162000 |
| C | 4.101798000 | 0.936224000 | 5.928120000 |
| H | 4.787391000 | 1.262566000 | 6.712321000 |
| C | 3.665526000 | -0.390746000 | 5.885269000 |
| H | 4.006852000 | -1.105210000 | 6.636801000 |
| C | 2.790162000 | -0.814591000 | 4.879681000 |
| H | 2.465160000 | -1.855360000 | 4.865372000 |
| C | 2.336152000 | 0.092844000 | 3.909488000 |
| C | -0.182060000 | 0.726834000 | 2.572033000 |
| H | -0.975268000 | 0.245106000 | 3.161820000 |
| H | 0.136990000 | 1.628641000 | 3.113594000 |
| C | -0.644419000 | 1.085541000 | 1.159624000 |
| H | -1.332883000 | 1.941115000 | 1.156910000 |
| H | -1.165620000 | 0.238702000 | 0.690579000 |
| C | 0.633573000 | 4.042248000 | 1.120816000 |
| H | -0.439142000 | 3.871417000 | 1.224339000 |
| C | 1.170656000 | 5.276012000 | 1.498400000 |
| H | 0.514509000 | 6.051206000 | 1.899239000 |
| C | 2.542165000 | 5.519483000 | 1.361205000 |
| H | 2.957253000 | 6.484921000 | 1.656904000 |
| C | 3.379245000 | 4.523110000 | 0.848036000 |
| H | 4.450231000 | 4.704850000 | 0.738299000 |
| C | 2.846681000 | 3.283288000 | 0.486672000 |
| H | 3.499549000 | 2.494540000 | 0.111346000 |
| C | 1.470275000 | 3.033847000 | 0.614897000 |
| C | 0.615926000 | 2.767004000 | -2.342037000 |
| H | 1.288189000 | 3.513018000 | -1.914745000 |
| C | 0.159676000 | 2.918452000 | -3.655353000 |

| | | | |
|---|---|---|---|
| H | 0.489817000 | 3.776387000 | -4.244381000 |
| C | -0.719731000 | 1.982672000 | -4.208266000 |
| H | -1.078550000 | 2.104904000 | -5.231815000 |
| C | -1.128346000 | 0.881916000 | -3.445998000 |
| H | -1.805045000 | 0.138061000 | -3.871461000 |
| C | -0.662655000 | 0.720792000 | -2.140097000 |
| H | -0.967605000 | -0.158055000 | -1.569577000 |
| C | 0.200434000 | 1.671640000 | -1.567784000 |
| C | 2.822106000 | -0.272794000 | -1.419516000 |
| H | 3.627774000 | -1.011929000 | -1.515562000 |
| H | 3.216260000 | 0.730399000 | -1.624122000 |
| H | 2.009260000 | -0.505469000 | -2.113719000 |
| N | 3.991900000 | 0.109666000 | 1.101989000 |
| C | 5.018545000 | 0.428638000 | 1.548465000 |
| C | 6.286774000 | 0.822842000 | 2.114875000 |
| H | 6.438141000 | 1.903905000 | 1.982302000 |
| H | 6.303187000 | 0.588163000 | 3.189229000 |
| H | 7.098745000 | 0.280285000 | 1.609844000 |
| H | -3.603785000 | 0.141251000 | 3.270485000 |
| C | -4.134613000 | 0.145289000 | 2.307781000 |
| C | -3.672747000 | -0.955131000 | 1.487664000 |
| H | -5.213443000 | 0.047540000 | 2.493514000 |
| N | -3.297601000 | -1.833344000 | 0.820367000 |
| H | -3.942761000 | 1.102464000 | 1.799362000 |
| H | -1.674450000 | 4.697469000 | -1.361751000 |
| C | -2.635270000 | 4.179236000 | -1.487333000 |
| C | -3.150114000 | 3.773901000 | -0.196563000 |
| H | -3.347226000 | 4.853234000 | -1.983712000 |
| N | -3.556194000 | 3.436598000 | 0.841969000 |
| H | -2.478924000 | 3.293932000 | -2.120008000 |
| H | -4.488705000 | 1.505153000 | -2.326429000 |
| N | -4.559109000 | -1.604634000 | -2.901142000 |
| C | -4.486515000 | -0.591942000 | -2.330565000 |
| C | -4.390414000 | 0.667315000 | -1.621955000 |
| H | -5.185064000 | 0.738360000 | -0.866359000 |
| H | -3.414671000 | 0.737402000 | -1.122168000 |
| H | 1.296705000 | -0.759841000 | -4.622584000 |
| C | 3.376882000 | -0.828771000 | -4.842285000 |
| C | 2.115143000 | -0.139525000 | -5.014182000 |
| N | 4.392590000 | -1.381786000 | -4.702778000 |
| H | 2.128810000 | 0.811100000 | -4.463661000 |
| H | 1.936066000 | 0.063507000 | -6.079194000 |
| H | 4.628435000 | 1.991298000 | -3.986761000 |
| C | 5.448928000 | 2.642429000 | -2.169724000 |
| C | 4.518579000 | 2.814503000 | -3.266782000 |
| N | 6.192767000 | 2.506622000 | -1.283760000 |
| H | 3.487597000 | 2.815524000 | -2.885062000 |
| H | 4.711324000 | 3.767408000 | -3.779219000 |
| H | 6.424332000 | -3.999579000 | -2.347035000 |
| C | 6.469840000 | -2.306258000 | -1.111783000 |
| C | 5.741974000 | -3.233341000 | -1.953108000 |
| N | 7.054149000 | -1.557479000 | -0.437254000 |
| H | 4.952329000 | -3.723438000 | -1.365979000 |
| H | 5.283082000 | -2.692935000 | -2.795327000 |

**PPH3-3Acn**

| | | | |
|---|---|---|---|
| P | 3.624020000 | 1.072450000 | -0.447044000 |
| C | 6.098667000 | 2.440657000 | -0.831682000 |
| H | 6.297516000 | 2.266619000 | 0.226783000 |
| C | 7.005924000 | 3.186447000 | -1.591935000 |
| H | 7.902611000 | 3.589651000 | -1.116928000 |
| C | 6.771718000 | 3.411276000 | -2.950922000 |
| H | 7.482345000 | 3.990249000 | -3.543323000 |
| C | 5.616360000 | 2.891216000 | -3.547069000 |
| H | 5.420535000 | 3.064740000 | -4.607072000 |
| C | 4.703394000 | 2.159625000 | -2.787057000 |
| H | 3.793440000 | 1.782461000 | -3.258273000 |
| C | 4.938609000 | 1.911690000 | -1.420372000 |
| C | 2.342418000 | -1.319667000 | -1.010087000 |
| H | 1.563284000 | -0.894102000 | -0.373162000 |
| C | 2.167988000 | -2.587831000 | -1.569285000 |
| H | 1.255965000 | -3.151042000 | -1.363452000 |
| C | 3.154797000 | -3.128086000 | -2.400800000 |
| H | 3.016197000 | -4.114417000 | -2.846715000 |
| C | 4.317585000 | -2.395853000 | -2.664462000 |

| | | | |
|---|---|---|---|
| H | 5.090377000 | -2.811424000 | -3.313971000 |
| C | 4.497610000 | -1.130520000 | -2.095519000 |
| H | 5.404752000 | -0.563347000 | -2.311518000 |
| C | 3.512583000 | -0.579615000 | -1.258446000 |
| C | 5.549850000 | -0.275284000 | 1.196246000 |
| H | 5.887935000 | -0.790322000 | 0.294918000 |
| C | 6.162009000 | -0.550762000 | 2.420231000 |
| H | 6.973416000 | -1.279810000 | 2.469889000 |
| C | 5.734249000 | 0.102698000 | 3.582205000 |
| H | 6.210410000 | -0.117478000 | 4.539425000 |
| C | 4.696757000 | 1.036331000 | 3.513688000 |
| H | 4.360347000 | 1.549267000 | 4.416705000 |
| C | 4.080794000 | 1.309673000 | 2.287779000 |
| H | 3.270453000 | 2.040056000 | 2.232270000 |
| C | 4.501256000 | 0.656217000 | 1.118599000 |
| N | 0.877346000 | 1.835848000 | -3.095298000 |
| H | 2.426819000 | -0.739457000 | -4.072920000 |
| C | 1.131212000 | 0.861730000 | -3.681003000 |
| C | 1.457680000 | -0.346477000 | -4.411746000 |
| H | 1.512243000 | -0.133724000 | -5.488054000 |
| H | 0.689984000 | -1.112120000 | -4.236505000 |
| N | 2.524932000 | -2.405939000 | 2.697542000 |
| C | 3.327414000 | -2.922648000 | 2.030611000 |
| H | 4.441592000 | -3.025473000 | 0.258796000 |
| C | 4.321585000 | -3.575547000 | 1.201926000 |
| H | 5.287544000 | -3.604162000 | 1.723838000 |
| H | 4.006467000 | -4.602906000 | 0.973515000 |
| H | 3.392306000 | 4.617738000 | -1.971430000 |
| C | 2.448874000 | 4.582146000 | -1.408985000 |
| C | 2.721811000 | 4.635409000 | 0.012978000 |
| N | 2.941141000 | 4.681385000 | 1.156082000 |
| H | 1.821512000 | 5.434034000 | -1.705623000 |
| H | 1.930007000 | 3.645579000 | -1.663660000 |

**Ni(Triphos)PPH3-3Acn**

| | | | |
|---|---|---|---|
| Ni | 0.373577000 | -0.026479000 | -1.843902000 |
| P | 0.617987000 | -1.877225000 | -0.743174000 |
| P | 2.428441000 | 0.511362000 | -1.391216000 |
| P | -0.299263000 | 1.690882000 | -0.708718000 |
| P | -0.602335000 | -0.266131000 | -3.768909000 |
| C | -1.595537000 | -1.799823000 | 0.972530000 |
| H | -1.646690000 | -0.756234000 | 0.657291000 |
| C | -2.523237000 | -2.303280000 | 1.889703000 |
| H | -3.291509000 | -1.643158000 | 2.297452000 |
| C | -2.466851000 | -3.647159000 | 2.275295000 |
| H | -3.192481000 | -4.045794000 | 2.986683000 |
| C | -1.474145000 | -4.480008000 | 1.744425000 |
| H | -1.419948000 | -5.529761000 | 2.042361000 |
| C | -0.543254000 | -3.972608000 | 0.833183000 |
| H | 0.225015000 | -4.629697000 | 0.420597000 |
| C | -0.593437000 | -2.624925000 | 0.439600000 |
| C | 0.179409000 | -3.885414000 | -2.626071000 |
| H | -0.799820000 | -3.411650000 | -2.723649000 |
| C | 0.494460000 | -4.984452000 | -3.423479000 |
| H | -0.240872000 | -5.364784000 | -4.135014000 |
| C | 1.758403000 | -5.580231000 | -3.332586000 |
| H | 2.011841000 | -6.431516000 | -3.967146000 |
| C | 2.694004000 | -5.074469000 | -2.426910000 |
| H | 3.682437000 | -5.531816000 | -2.346721000 |
| C | 2.369557000 | -3.982367000 | -1.613559000 |
| H | 3.113194000 | -3.617437000 | -0.903992000 |
| C | 1.108375000 | -3.372264000 | -1.702236000 |
| C | 2.064546000 | -1.598885000 | 0.406974000 |
| H | 1.640788000 | -1.003843000 | 1.231642000 |
| H | 2.429107000 | -2.541859000 | 0.843116000 |
| C | 3.172999000 | -0.822995000 | -0.307601000 |
| H | 3.743939000 | -1.495413000 | -0.961476000 |
| H | 3.886580000 | -0.372176000 | 0.398438000 |
| C | 4.466677000 | -0.179571000 | -3.202317000 |
| H | 4.247589000 | -1.213382000 | -2.926676000 |
| C | 5.394437000 | 0.059515000 | -4.216900000 |
| H | 5.894011000 | -0.783750000 | -4.698561000 |
| C | 5.677472000 | 1.369034000 | -4.621401000 |

| | | | |
|---|---|---|---|
| H | 6.399311000 | 1.556940000 | -5.418012000 |
| C | 5.024081000 | 2.433534000 | -3.993169000 |
| H | 5.234392000 | 3.461149000 | -4.297167000 |
| C | 4.099147000 | 2.195114000 | -2.972920000 |
| H | 3.602401000 | 3.048525000 | -2.509957000 |
| C | 3.805189000 | 0.883319000 | -2.551651000 |
| C | 2.409035000 | 1.984910000 | -0.236563000 |
| H | 3.266008000 | 1.935991000 | 0.453245000 |
| H | 2.525960000 | 2.892950000 | -0.844044000 |
| C | 1.071368000 | 2.051236000 | 0.508809000 |
| H | 0.934520000 | 3.031566000 | 0.990111000 |
| H | 1.013962000 | 1.281417000 | 1.292513000 |
| C | 0.496509000 | 3.536823000 | -2.651940000 |
| H | 1.084640000 | 2.695746000 | -3.026472000 |
| C | 0.572614000 | 4.783709000 | -3.274587000 |
| H | 1.244763000 | 4.923959000 | -4.123311000 |
| C | -0.233596000 | 5.837484000 | -2.832839000 |
| H | -0.186762000 | 6.809764000 | -3.327378000 |
| C | -1.112581000 | 5.635655000 | -1.762964000 |
| H | -1.750722000 | 6.452293000 | -1.418129000 |
| C | -1.171064000 | 4.393118000 | -1.124116000 |
| H | -1.850120000 | 4.247836000 | -0.281596000 |
| C | -0.364090000 | 3.330334000 | -1.561078000 |
| C | -1.822946000 | 1.876699000 | 1.710569000 |
| H | -0.894322000 | 2.062648000 | 2.251560000 |
| C | -3.015147000 | 1.751444000 | 2.433183000 |
| H | -3.001471000 | 1.853619000 | 3.520609000 |
| C | -4.220036000 | 1.496920000 | 1.772579000 |
| H | -5.147364000 | 1.397353000 | 2.339746000 |
| C | -4.229882000 | 1.361708000 | 0.377937000 |
| H | -5.164867000 | 1.154701000 | -0.146812000 |
| C | -3.040359000 | 1.467238000 | -0.341586000 |
| H | -3.044821000 | 1.313768000 | -1.423480000 |
| C | -1.821813000 | 1.731536000 | 0.312954000 |
| C | 0.100437000 | 1.576440000 | -5.825925000 |
| H | 0.919883000 | 0.895370000 | -6.063607000 |
| C | -0.027571000 | 2.775357000 | -6.534362000 |
| H | 0.695506000 | 3.019448000 | -7.315739000 |
| C | -1.078058000 | 3.653539000 | -6.252119000 |
| H | -1.178748000 | 4.587998000 | -6.807281000 |
| C | -1.995421000 | 3.327964000 | -5.247560000 |
| H | -2.813249000 | 4.011279000 | -5.009467000 |
| C | -1.860520000 | 2.137908000 | -4.532562000 |
| H | -2.577890000 | 1.901630000 | -3.744355000 |
| C | -0.818596000 | 1.238171000 | -4.820075000 |
| C | -2.802878000 | -1.508321000 | -2.561405000 |
| H | -2.124803000 | -1.576172000 | -1.707812000 |
| C | -4.106948000 | -2.003317000 | -2.470316000 |
| H | -4.443009000 | -2.477306000 | -1.545534000 |
| C | -4.978873000 | -1.876342000 | -3.555515000 |
| H | -6.000911000 | -2.254070000 | -3.486874000 |
| C | -4.539324000 | -1.251301000 | -4.728614000 |
| H | -5.218032000 | -1.141835000 | -5.577033000 |
| C | -3.234050000 | -0.760854000 | -4.817458000 |
| H | -2.908182000 | -0.258617000 | -5.730123000 |
| C | -2.346183000 | -0.890395000 | -3.734717000 |
| C | -0.453369000 | -2.259688000 | -5.860398000 |
| H | -1.543683000 | -2.275000000 | -5.883679000 |
| C | 0.263301000 | -3.104316000 | -6.713897000 |
| H | -0.275397000 | -3.765912000 | -7.395780000 |
| C | 1.661544000 | -3.106975000 | -6.695673000 |
| H | 2.217663000 | -3.767847000 | -7.363484000 |
| C | 2.342121000 | -2.266443000 | -5.808380000 |
| H | 3.434067000 | -2.264400000 | -5.778530000 |
| C | 1.624531000 | -1.440446000 | -4.941286000 |
| H | 2.148733000 | -0.811202000 | -4.217974000 |
| C | 0.220441000 | -1.416970000 | -4.964256000 |
| N | 3.643137000 | -0.077193000 | 6.110514000 |
| C | 3.732086000 | 0.109802000 | 4.964553000 |
| C | 3.822602000 | 0.338595000 | 3.536248000 |
| H | 4.595311000 | 1.086405000 | 3.315072000 |
| H | 4.072354000 | -0.598712000 | 3.019707000 |
| H | 2.856473000 | 0.709439000 | 3.168025000 |
| N | 0.608573000 | 1.470141000 | 4.218876000 |
| C | 0.535301000 | 1.462627000 | 5.381309000 |
| C | 0.453629000 | 1.433781000 | 6.827279000 |

| | | | |
|---|---|---|---|
| H | 1.439098000 | 1.176310000 | 7.240581000 |
| H | 0.141516000 | 2.414188000 | 7.211848000 |
| H | -0.272850000 | 0.670779000 | 7.139662000 |
| N | -1.030203000 | -1.717135000 | 6.109277000 |
| H | 1.514485000 | -2.286225000 | 4.315323000 |
| C | -0.356031000 | -1.878130000 | 5.173236000 |
| H | 0.098840000 | -2.883092000 | 3.388115000 |
| C | 0.483832000 | -2.062404000 | 4.007441000 |
| H | 0.477259000 | -1.142494000 | 3.407985000 |

**CH3NiPPH3Acn-3Acn**

| | | | |
|---|---|---|---|
| Ni | 2.256221000 | -0.227481000 | 0.625035000 |
| P | 1.262518000 | -2.163789000 | 0.426219000 |
| P | 1.270820000 | -0.086986000 | 2.594426000 |
| P | 1.234283000 | 1.668782000 | 0.155950000 |
| C | -0.894741000 | -2.179878000 | -1.395871000 |
| H | -1.492314000 | -1.696845000 | -0.620870000 |
| C | -1.471897000 | -2.394989000 | -2.651074000 |
| H | -2.502759000 | -2.078870000 | -2.825680000 |
| C | -0.729009000 | -2.992388000 | -3.673909000 |
| H | -1.179217000 | -3.154894000 | -4.654869000 |
| C | 0.592387000 | -3.384383000 | -3.434994000 |
| H | 1.177843000 | -3.852380000 | -4.228569000 |
| C | 1.171519000 | -3.175798000 | -2.181710000 |
| H | 2.201454000 | -3.489154000 | -2.002926000 |
| C | 0.431958000 | -2.575566000 | -1.146536000 |
| C | 3.608459000 | -3.471472000 | 1.213269000 |
| H | 4.008279000 | -2.460118000 | 1.307316000 |
| C | 4.395630000 | -4.582699000 | 1.532959000 |
| H | 5.421314000 | -4.440243000 | 1.878211000 |
| C | 3.869003000 | -5.870791000 | 1.408601000 |
| H | 4.482380000 | -6.738913000 | 1.656659000 |
| C | 2.553216000 | -6.049362000 | 0.962700000 |
| H | 2.140836000 | -7.055194000 | 0.863727000 |
| C | 1.766318000 | -4.942451000 | 0.642253000 |
| H | 0.743779000 | -5.087890000 | 0.287237000 |
| C | 2.290914000 | -3.644200000 | 0.767695000 |
| C | -0.074726000 | -2.290694000 | 1.708890000 |
| H | -0.945601000 | -1.719879000 | 1.356237000 |
| H | -0.384610000 | -3.337739000 | 1.834287000 |
| C | 0.465912000 | -1.696536000 | 3.008514000 |
| H | 1.238197000 | -2.355801000 | 3.430434000 |
| H | -0.318048000 | -1.556873000 | 3.766707000 |
| C | 2.774845000 | 1.708602000 | 4.094650000 |
| H | 2.453205000 | 2.451048000 | 3.361771000 |
| C | 3.668472000 | 2.087242000 | 5.096654000 |
| H | 4.018093000 | 3.120098000 | 5.143816000 |
| C | 4.123060000 | 1.145264000 | 6.026173000 |
| H | 4.825104000 | 1.440624000 | 6.807624000 |
| C | 3.675373000 | -0.176177000 | 5.947657000 |
| H | 4.025769000 | -0.917539000 | 6.667797000 |
| C | 2.778772000 | -0.559659000 | 4.945829000 |
| H | 2.450394000 | -1.598118000 | 4.901639000 |
| C | 2.309646000 | 0.382320000 | 4.016396000 |
| C | -0.092285000 | 1.184102000 | 2.540419000 |
| H | -0.991502000 | 0.761838000 | 3.011437000 |
| H | 0.233635000 | 2.031775000 | 3.158091000 |
| C | -0.358533000 | 1.622828000 | 1.099934000 |
| H | -0.895697000 | 2.581272000 | 1.052392000 |
| H | -0.979315000 | 0.879711000 | 0.579575000 |
| C | 1.461249000 | 4.192892000 | 1.476282000 |
| H | 0.386572000 | 4.158314000 | 1.660606000 |
| C | 2.202273000 | 5.267542000 | 1.977550000 |
| H | 1.699586000 | 6.049874000 | 2.548974000 |
| C | 3.579361000 | 5.339884000 | 1.748806000 |
| H | 4.156149000 | 6.177197000 | 2.145280000 |
| C | 4.216046000 | 4.335503000 | 1.011041000 |
| H | 5.290811000 | 4.387070000 | 0.827560000 |
| C | 3.480873000 | 3.254606000 | 0.523102000 |
| H | 3.983850000 | 2.457296000 | -0.026340000 |
| C | 2.095391000 | 3.171515000 | 0.750204000 |
| C | 0.798032000 | 3.394807000 | -2.063364000 |
| H | 1.236084000 | 4.193776000 | -1.463153000 |
| C | 0.334799000 | 3.666772000 | -3.354432000 |
| H | 0.416437000 | 4.679097000 | -3.754558000 |

| | | | |
|---|---|---|---|
| C | -0.232786000 | 2.650420000 | -4.129581000 |
| H | -0.593557000 | 2.867452000 | -5.136499000 |
| C | -0.333408000 | 1.354603000 | -3.611753000 |
| H | -0.774802000 | 0.553427000 | -4.207877000 |
| C | 0.135043000 | 1.078322000 | -2.326780000 |
| H | 0.056374000 | 0.065037000 | -1.929935000 |
| C | 0.694833000 | 2.097344000 | -1.537833000 |
| C | 3.012318000 | -0.437542000 | -1.224114000 |
| H | 3.659814000 | -1.325415000 | -1.215066000 |
| H | 3.617420000 | 0.456933000 | -1.423238000 |
| H | 2.237455000 | -0.543940000 | -1.990051000 |
| N | 4.045387000 | 0.029618000 | 1.346814000 |
| C | 5.031629000 | 0.326618000 | 1.889277000 |
| C | 6.233732000 | 0.714598000 | 2.591175000 |
| H | 6.509939000 | 1.744302000 | 2.321825000 |
| H | 6.055515000 | 0.668838000 | 3.675493000 |
| H | 7.064583000 | 0.042937000 | 2.334425000 |
| H | -4.071839000 | 0.970535000 | 3.591316000 |
| C | -4.641560000 | 0.595432000 | 2.730048000 |
| C | -3.751071000 | -0.043513000 | 1.783772000 |
| H | -5.393853000 | -0.122395000 | 3.084809000 |
| N | -3.038642000 | -0.546580000 | 1.012115000 |
| H | -5.148683000 | 1.436877000 | 2.236285000 |
| H | -2.410915000 | 2.816164000 | -1.513682000 |
| C | -3.171032000 | 2.034307000 | -1.647484000 |
| C | -4.322007000 | 2.296205000 | -0.808044000 |
| H | -3.469541000 | 2.003741000 | -2.703575000 |
| N | -5.246532000 | 2.491649000 | -0.126967000 |
| H | -2.737150000 | 1.061743000 | -1.380097000 |
| H | -7.426700000 | -1.280823000 | -0.920672000 |
| N | -4.766796000 | -0.941511000 | -2.604020000 |
| C | -5.513906000 | -0.930817000 | -1.710853000 |
| C | -6.444025000 | -0.909533000 | -0.599418000 |
| H | -6.071279000 | -1.546725000 | 0.214126000 |
| H | -6.551685000 | 0.119332000 | -0.227921000 |

**Co(dmgBF2)2py-3Acn**

| | | | |
|---|---|---|---|
| Co | 2.926131000 | -0.628302000 | -0.353738000 |
| N | 1.661736000 | 0.544405000 | 0.348047000 |
| N | 1.705428000 | -1.980631000 | -0.728990000 |
| N | 4.070299000 | 0.472739000 | 0.623640000 |
| N | 4.105351000 | -2.061512000 | -0.431132000 |
| C | 2.195338000 | -3.191776000 | -1.016209000 |
| C | 3.621069000 | -3.241957000 | -0.833661000 |
| C | 2.110444000 | 1.594623000 | 1.040158000 |
| C | 3.540870000 | 1.557761000 | 1.195474000 |
| O | 5.427597000 | 0.306625000 | 0.783495000 |
| O | 5.456220000 | -2.029117000 | -0.149545000 |
| O | 0.294040000 | 0.429184000 | 0.242068000 |
| O | 0.337822000 | -1.854973000 | -0.811373000 |
| C | 1.314963000 | -4.324607000 | -1.428700000 |
| C | 4.493286000 | -4.441468000 | -1.004874000 |
| C | 1.196474000 | 2.618869000 | 1.625306000 |
| C | 4.359983000 | 2.551075000 | 1.948122000 |
| B | 6.073109000 | -0.664541000 | -0.151742000 |
| B | -0.185129000 | -0.454544000 | -0.864288000 |
| H | 1.743929000 | 3.527034000 | 1.902145000 |
| H | 0.697445000 | 2.238682000 | 2.532162000 |
| H | 0.405670000 | 2.880449000 | 0.909190000 |
| H | 3.841781000 | 3.514035000 | 2.027012000 |
| H | 4.564131000 | 2.194293000 | 2.971314000 |
| H | 5.326453000 | 2.703830000 | 1.450032000 |
| H | 0.709562000 | -4.045156000 | -2.303905000 |
| H | 1.901727000 | -5.215461000 | -1.676935000 |
| H | 0.607084000 | -4.588972000 | -0.627753000 |
| H | 3.951222000 | -5.263483000 | -1.485020000 |
| H | 4.871560000 | -4.802000000 | -0.034590000 |
| H | 5.373245000 | -4.193572000 | -1.615675000 |
| F | 0.091469000 | 0.131401000 | -2.122412000 |
| F | -1.572733000 | -0.571789000 | -0.670166000 |
| F | 6.115501000 | -0.141769000 | -1.463890000 |
| F | 7.373320000 | -0.849862000 | 0.351511000 |
| N | 3.159154000 | 0.148997000 | -2.187814000 |

| | | | |
|---|---|---|---|
| C | 3.228012000 | 1.484300000 | -2.366770000 |
| C | 3.375982000 | 2.059551000 | -3.625117000 |
| C | 3.458173000 | 1.230766000 | -4.746788000 |
| C | 3.389921000 | -0.151872000 | -4.558402000 |
| C | 3.240694000 | -0.653493000 | -3.268980000 |
| H | 3.160966000 | 2.095376000 | -1.463867000 |
| H | 3.424956000 | 3.144966000 | -3.716467000 |
| H | 3.573439000 | 1.653171000 | -5.745752000 |
| H | 3.450139000 | -0.842971000 | -5.399592000 |
| H | 3.182795000 | -1.727283000 | -3.076616000 |
| H | 5.392736000 | -0.878890000 | 3.329310000 |
| H | 5.525017000 | -2.248122000 | 2.200742000 |
| C | 5.669065000 | -1.938953000 | 3.247112000 |
| C | 4.848266000 | -2.738552000 | 4.131432000 |
| N | 4.172882000 | -3.382211000 | 4.829333000 |
| H | 6.730454000 | -2.051283000 | 3.507228000 |
| H | 0.745777000 | -0.565018000 | 3.116193000 |
| H | 1.665493000 | -1.940888000 | 3.793037000 |
| C | 1.752330000 | -0.972900000 | 3.282470000 |
| C | 2.541281000 | -0.063736000 | 4.085976000 |
| N | 3.189439000 | 0.657140000 | 4.732789000 |
| H | 2.227937000 | -1.115306000 | 2.295114000 |
| H | 2.757145000 | -3.939545000 | 1.568190000 |
| H | 2.353332000 | -5.669262000 | 1.757299000 |
| C | 2.239276000 | -4.672085000 | 2.204034000 |
| H | 2.704321000 | -4.664153000 | 3.199919000 |
| C | 0.835798000 | -4.324776000 | 2.302164000 |
| N | -0.289497000 | -4.035020000 | 2.381575000 |

**CH3Co(DmgBF2)Py-3Acn**

| | | | |
|---|---|---|---|
| Co | 3.005556000 | -0.705353000 | -0.431193000 |
| N | 1.758755000 | 0.576214000 | 0.156352000 |
| N | 1.778240000 | -1.931073000 | -1.166752000 |
| N | 4.203422000 | 0.419936000 | 0.483469000 |
| N | 4.224160000 | -2.083310000 | -0.833814000 |
| C | 2.268995000 | -3.085885000 | -1.553666000 |
| C | 3.716259000 | -3.175223000 | -1.358523000 |
| C | 2.237514000 | 1.572291000 | 0.867800000 |
| C | 3.682160000 | 1.475334000 | 1.068417000 |
| O | 5.511024000 | 0.143873000 | 0.697919000 |
| O | 5.529587000 | -2.085025000 | -0.477637000 |
| O | 0.416993000 | 0.467229000 | 0.013660000 |
| O | 0.435349000 | -1.764041000 | -1.164461000 |
| C | 1.458410000 | -4.209736000 | -2.094270000 |
| C | 4.496874000 | -4.400752000 | -1.675613000 |
| C | 1.419978000 | 2.681662000 | 1.431039000 |
| C | 4.447306000 | 2.446601000 | 1.896056000 |
| B | 6.180715000 | -0.732698000 | -0.337800000 |
| B | -0.057303000 | -0.338369000 | -1.176390000 |
| H | 1.759273000 | 3.646150000 | 1.023378000 |
| H | 1.535439000 | 2.731199000 | 2.523620000 |
| H | 0.362611000 | 2.544891000 | 1.185322000 |
| H | 4.165970000 | 3.477422000 | 1.640764000 |
| H | 4.215693000 | 2.297343000 | 2.963058000 |
| H | 5.523683000 | 2.313590000 | 1.747677000 |
| H | 0.417751000 | -3.901119000 | -2.232365000 |
| H | 1.867588000 | -4.550502000 | -3.056554000 |
| H | 1.496025000 | -5.062204000 | -1.397946000 |
| H | 4.258839000 | -4.757951000 | -2.687402000 |
| H | 4.227112000 | -5.203214000 | -0.970495000 |
| H | 5.570544000 | -4.202630000 | -1.601079000 |
| F | 0.312029000 | 0.298746000 | -2.365707000 |
| F | -1.436200000 | -0.429716000 | -1.020277000 |
| F | 6.219376000 | -0.075013000 | -1.571932000 |
| F | 7.447602000 | -0.988836000 | 0.175328000 |
| C | 2.653803000 | -1.627908000 | 1.326522000 |
| H | 2.860173000 | -0.925682000 | 2.140418000 |
| H | 1.599518000 | -1.927160000 | 1.336707000 |
| H | 3.308140000 | -2.502098000 | 1.405300000 |
| N | 3.311505000 | 0.237033000 | -2.233114000 |
| C | 3.401096000 | 1.578973000 | -2.303427000 |
| C | 3.594794000 | 2.255072000 | -3.503893000 |
| C | 3.701507000 | 1.521983000 | -4.685668000 |
| C | 3.610529000 | 0.131967000 | -4.613448000 |

| | | | |
|---|---|---|---|
| C | 3.416000000 | -0.470534000 | -3.374566000 |
| H | 3.315625000 | 2.128484000 | -1.367901000 |
| H | 3.658584000 | 3.342899000 | -3.499317000 |
| H | 3.852973000 | 2.023141000 | -5.642382000 |
| H | 3.687549000 | -0.491605000 | -5.503777000 |
| H | 3.341565000 | -1.553281000 | -3.292651000 |
| H | 4.544272000 | -0.129485000 | 4.281570000 |
| H | 5.684034000 | -0.520008000 | 2.956322000 |
| C | 5.408155000 | -0.749572000 | 3.996506000 |
| C | 5.059143000 | -2.149854000 | 4.116815000 |
| N | 4.768620000 | -3.273932000 | 4.210472000 |
| H | 6.255718000 | -0.512355000 | 4.654014000 |
| H | -0.081867000 | -0.092944000 | 2.475455000 |
| H | -0.858628000 | 0.223764000 | 4.056004000 |
| C | -0.009965000 | -0.266062000 | 3.559469000 |
| C | 1.238528000 | 0.266116000 | 4.067758000 |
| N | 2.245226000 | 0.699367000 | 4.462216000 |
| H | -0.061936000 | -1.346948000 | 3.749051000 |
| N | 2.458718000 | -5.850875000 | 0.831659000 |
| C | 2.186336000 | -5.372340000 | 1.858076000 |
| C | 1.856367000 | -4.776149000 | 3.136406000 |
| H | 1.106549000 | -3.984881000 | 2.998675000 |
| H | 1.448637000 | -5.538843000 | 3.814057000 |
| H | 2.760834000 | -4.337661000 | 3.586110000 |

**CH3Co(DmgBF2)2Py**

| | | | |
|---|---|---|---|
| Co | 2.613050000 | 0.028471000 | 0.022098000 |
| N | 2.673450000 | 1.031977000 | 1.613511000 |
| N | 2.660940000 | 1.474039000 | -1.181817000 |
| N | 2.347359000 | -1.386837000 | 1.229173000 |
| N | 2.338829000 | -0.944740000 | -1.561118000 |
| C | 2.460879000 | 1.184649000 | -2.449098000 |
| C | 2.265939000 | -0.246851000 | -2.671841000 |
| C | 2.481560000 | 0.364668000 | 2.730287000 |
| C | 2.285018000 | -1.065770000 | 2.501509000 |
| O | 2.057213000 | -2.658876000 | 0.875196000 |
| O | 2.055046000 | -2.265048000 | -1.618522000 |
| O | 2.755834000 | 2.380527000 | 1.670309000 |
| O | 2.739194000 | 2.774467000 | -0.819138000 |
| C | 2.400472000 | 2.196956000 | -3.538997000 |
| C | 1.967091000 | -0.856674000 | -3.996194000 |
| C | 2.432949000 | 0.986102000 | 4.082307000 |
| C | 1.995581000 | -2.049500000 | 3.580164000 |
| B | 2.578162000 | -3.103045000 | -0.473043000 |
| B | 3.423709000 | 3.056193000 | 0.496779000 |
| H | 3.164138000 | 0.510522000 | 4.752348000 |
| H | 1.437845000 | 0.850097000 | 4.532901000 |
| H | 2.649340000 | 2.056694000 | 4.018191000 |
| H | 2.394918000 | -1.709103000 | 4.542267000 |
| H | 0.907599000 | -2.184722000 | 3.692675000 |
| H | 2.425213000 | -3.026661000 | 3.327420000 |
| H | 2.682638000 | 3.183286000 | -3.158186000 |
| H | 3.074666000 | 1.920540000 | -4.362119000 |
| H | 1.382232000 | 2.256276000 | -3.953790000 |
| H | 2.278723000 | -0.199032000 | -4.814880000 |
| H | 0.885124000 | -1.039760000 | -4.096488000 |
| H | 2.472815000 | -1.826319000 | -4.089980000 |
| F | 4.775112000 | 2.697247000 | 0.430683000 |
| F | 3.243067000 | 4.418435000 | 0.713827000 |
| F | 3.977290000 | -3.108468000 | -0.475173000 |
| F | 2.035627000 | -4.367545000 | -0.672264000 |
| C | 0.599355000 | 0.209940000 | 0.057663000 |
| H | 0.298959000 | 0.611796000 | 1.031731000 |
| H | 0.291872000 | 0.885534000 | -0.748304000 |
| H | 0.177700000 | -0.790376000 | -0.094746000 |
| N | 4.647917000 | -0.231117000 | -0.026152000 |
| C | 5.335543000 | -0.503402000 | 1.099376000 |
| C | 6.713974000 | -0.692031000 | 1.106740000 |
| C | 7.418207000 | -0.597596000 | -0.093590000 |
| C | 6.706047000 | -0.316311000 | -1.259515000 |
| C | 5.327893000 | -0.140636000 | -1.185166000 |
| H | 4.762367000 | -0.571849000 | 2.022161000 |
| H | 7.217936000 | -0.909685000 | 2.048109000 |
| H | 8.499104000 | -0.740229000 | -0.119871000 |

| | | | |
|---|---|---|---|
| H | 7.203606000 | -0.231203000 | -2.225280000 |
| H | 4.748637000 | 0.079897000 | -2.079962000 |

**CH3Co(DmgBF2)2Acn**

| | | | |
|---|---|---|---|
| Co | 0.465236000 | -0.130739000 | 8.070055000 |
| N | 0.205499000 | -1.997056000 | 8.029839000 |
| N | -1.349228000 | 0.370461000 | 7.987971000 |
| N | 2.279252000 | -0.644795000 | 7.985355000 |
| N | 0.715181000 | 1.737417000 | 7.946400000 |
| C | -1.593905000 | 1.655371000 | 7.897675000 |
| C | -0.373739000 | 2.463695000 | 7.866365000 |
| C | 1.289088000 | -2.733270000 | 7.976706000 |
| C | 2.515310000 | -1.933972000 | 7.944190000 |
| O | 3.308144000 | 0.220130000 | 7.841153000 |
| O | 1.919631000 | 2.335604000 | 7.808491000 |
| O | -0.997236000 | -2.605753000 | 7.930726000 |
| O | -2.384802000 | -0.492941000 | 7.893555000 |
| C | -2.963683000 | 2.225448000 | 7.777912000 |
| C | -0.385918000 | 3.945159000 | 7.719875000 |
| C | 1.272822000 | -4.219533000 | 7.895547000 |
| C | 3.872443000 | -2.537146000 | 7.841240000 |
| B | 3.099360000 | 1.605500000 | 8.409614000 |
| B | -2.174452000 | -1.856307000 | 8.511691000 |
| H | 2.169142000 | -4.647403000 | 8.360280000 |
| H | 1.251218000 | -4.546399000 | 6.843214000 |
| H | 0.376871000 | -4.616439000 | 8.387146000 |
| H | 4.064777000 | -3.198622000 | 8.699314000 |
| H | 3.954630000 | -3.149793000 | 6.930950000 |
| H | 4.637223000 | -1.755139000 | 7.815268000 |
| H | -3.687352000 | 1.586054000 | 8.297329000 |
| H | -3.003352000 | 3.239927000 | 8.191632000 |
| H | -3.265644000 | 2.281076000 | 6.719368000 |
| H | -0.863623000 | 4.234112000 | 6.771383000 |
| H | 0.634777000 | 4.338851000 | 7.738347000 |
| H | -0.965201000 | 4.408206000 | 8.532313000 |
| F | -2.039241000 | -1.742350000 | 9.900030000 |
| F | -3.291979000 | -2.597278000 | 8.141889000 |
| F | 2.973300000 | 1.543513000 | 9.802101000 |
| F | 4.214608000 | 2.331686000 | 8.004660000 |
| C | 0.516086000 | -0.130404000 | 6.065867000 |
| H | 0.578833000 | -1.166423000 | 5.713725000 |
| H | -0.394798000 | 0.349319000 | 5.689827000 |
| H | 1.405100000 | 0.435416000 | 5.763097000 |
| N | 0.472377000 | -0.091507000 | 10.030727000 |
| C | 0.487967000 | -0.056089000 | 11.188200000 |
| C | 0.509556000 | -0.008473000 | 12.630764000 |
| H | 1.110758000 | 0.847735000 | 12.966816000 |
| H | 0.949426000 | -0.933359000 | 13.028713000 |
| H | -0.513636000 | 0.096134000 | 13.017184000 |

**CH3Ni(Triphos)Acn-Co(dmgBF2)2Py**

| | | | |
|---|---|---|---|
| Ni | 2.625181000 | 0.288246000 | 0.478417000 |
| P | 1.745112000 | -1.613568000 | -0.169721000 |
| P | 1.410450000 | 0.042615000 | 2.314279000 |
| P | 1.637134000 | 2.224094000 | 0.278083000 |
| C | 0.685404000 | -0.948326000 | -2.643329000 |
| H | 0.494769000 | 0.020193000 | -2.176928000 |
| C | 0.281915000 | -1.172685000 | -3.959703000 |
| H | -0.231894000 | -0.381059000 | -4.508426000 |
| C | 0.538129000 | -2.406040000 | -4.569206000 |
| H | 0.226946000 | -2.581923000 | -5.600422000 |
| C | 1.197472000 | -3.411604000 | -3.855239000 |
| H | 1.401048000 | -4.374381000 | -4.327780000 |
| C | 1.594094000 | -3.193337000 | -2.532042000 |
| H | 2.099717000 | -3.984991000 | -1.976764000 |

| | | | |
|---|---|---|---|
| C | 1.334407000 | -1.960066000 | -1.915840000 |
| C | 4.054714000 | -3.102125000 | 0.245802000 |
| H | 4.546317000 | -2.255829000 | -0.236325000 |
| C | 4.817007000 | -4.171021000 | 0.718477000 |
| H | 5.900903000 | -4.162212000 | 0.589648000 |
| C | 4.194792000 | -5.241753000 | 1.370195000 |
| H | 4.791634000 | -6.070962000 | 1.753912000 |
| C | 2.806239000 | -5.245179000 | 1.529762000 |
| H | 2.314262000 | -6.078159000 | 2.035188000 |
| C | 2.039154000 | -4.182583000 | 1.042434000 |
| H | 0.956142000 | -4.210179000 | 1.170018000 |
| C | 2.656556000 | -3.097032000 | 0.399606000 |
| C | 0.089612000 | -1.729728000 | 0.641110000 |
| H | -0.548474000 | -1.004235000 | 0.114077000 |
| H | -0.361436000 | -2.718989000 | 0.488091000 |
| C | 0.218639000 | -1.370839000 | 2.118532000 |
| H | 0.621303000 | -2.215509000 | 2.694359000 |
| H | -0.750260000 | -1.099910000 | 2.561696000 |
| C | 2.928748000 | -1.570775000 | 3.983514000 |
| H | 2.791084000 | -2.332066000 | 3.212162000 |
| C | 3.734250000 | -1.856601000 | 5.085902000 |
| H | 4.198896000 | -2.840325000 | 5.173501000 |
| C | 3.955375000 | -0.882609000 | 6.066196000 |
| H | 4.588386000 | -1.105208000 | 6.926746000 |
| C | 3.363424000 | 0.376572000 | 5.937217000 |
| H | 3.531984000 | 1.142124000 | 6.696560000 |
| C | 2.553251000 | 0.666273000 | 4.835218000 |
| H | 2.108342000 | 1.658134000 | 4.753219000 |
| C | 2.316965000 | -0.309259000 | 3.854221000 |
| C | 0.365432000 | 1.540995000 | 2.596301000 |
| H | -0.544707000 | 1.273463000 | 3.152404000 |
| H | 0.945626000 | 2.254783000 | 3.198076000 |
| C | 0.049880000 | 2.149450000 | 1.230416000 |
| H | -0.385881000 | 3.153408000 | 1.321178000 |
| H | -0.666263000 | 1.517270000 | 0.683219000 |
| C | 1.912327000 | 4.961601000 | 0.945638000 |
| H | 1.026540000 | 5.129400000 | 0.328902000 |
| C | 2.506173000 | 6.032209000 | 1.615220000 |
| H | 2.080033000 | 7.033080000 | 1.523944000 |
| C | 3.645395000 | 5.823547000 | 2.403249000 |
| H | 4.107469000 | 6.663176000 | 2.925686000 |
| C | 4.190010000 | 4.542069000 | 2.517878000 |
| H | 5.078603000 | 4.376194000 | 3.129832000 |
| C | 3.597503000 | 3.467631000 | 1.846000000 |
| H | 4.014930000 | 2.462442000 | 1.928363000 |
| C | 2.456093000 | 3.670008000 | 1.058174000 |
| C | 2.118061000 | 3.553655000 | -2.134721000 |
| H | 3.080968000 | 3.821874000 | -1.696138000 |
| C | 1.823959000 | 3.933167000 | -3.445445000 |
| H | 2.563020000 | 4.489544000 | -4.025040000 |
| C | 0.589224000 | 3.601001000 | -4.014361000 |
| H | 0.362081000 | 3.898540000 | -5.039728000 |
| C | -0.353754000 | 2.892947000 | -3.263747000 |
| H | -1.325667000 | 2.631642000 | -3.685817000 |
| C | -0.061210000 | 2.509651000 | -1.951991000 |
| H | -0.816897000 | 1.961253000 | -1.389395000 |
| C | 1.175068000 | 2.839907000 | -1.373080000 |
| C | 3.536227000 | 0.558230000 | -1.292657000 |
| H | 4.173725000 | -0.316327000 | -1.479409000 |
| H | 4.162631000 | 1.456097000 | -1.201573000 |
| H | 2.819122000 | 0.680754000 | -2.111232000 |
| H | -3.109721000 | 4.723176000 | 2.599307000 |
| H | -1.930988000 | 3.619170000 | 3.341325000 |
| H | -3.643587000 | 3.468259000 | 3.751639000 |
| H | -3.100379000 | 5.193423000 | 0.580437000 |
| C | -2.950589000 | 3.690070000 | 2.927982000 |
| C | -3.044687000 | 4.414353000 | -0.187731000 |
| H | -2.075129000 | 4.508771000 | -0.703633000 |
| H | -3.825009000 | 4.599848000 | -0.939719000 |
| C | -3.153023000 | 2.726861000 | 1.805811000 |
| C | -3.199615000 | 3.053894000 | 0.405618000 |
| O | -3.123012000 | 0.980516000 | 3.309528000 |
| N | -3.261364000 | 1.409562000 | 2.005107000 |
| H | -5.734714000 | 2.317337000 | 1.022498000 |
| H | -8.233065000 | 2.360194000 | 1.062395000 |
| F | -3.324907000 | -0.665138000 | 4.912512000 |

| | | | |
|---|---|---|---|
| B | -3.733125000 | -0.351732000 | 3.603811000 |
| N | -3.339814000 | 1.959839000 | -0.350283000 |
| F | -5.143318000 | -0.279255000 | 3.525524000 |
| C | -6.317800000 | 1.416953000 | 0.815997000 |
| C | -7.709116000 | 1.431008000 | 0.836667000 |
| O | -3.338339000 | 2.165671000 | -1.711107000 |
| Co | -3.603923000 | 0.338843000 | 0.525455000 |
| N | -5.611691000 | 0.300710000 | 0.542469000 |
| O | -3.220596000 | -1.475461000 | 2.757012000 |
| C | -8.405494000 | 0.250173000 | 0.567670000 |
| H | -9.495982000 | 0.230500000 | 0.577263000 |
| N | -3.267130000 | -1.270202000 | 1.395036000 |
| B | -3.817395000 | 1.020338000 | -2.546393000 |
| F | -5.222350000 | 0.889330000 | -2.445361000 |
| F | -3.448522000 | 1.352165000 | -3.864776000 |
| C | -6.281775000 | -0.840476000 | 0.281355000 |
| C | -7.671835000 | -0.904765000 | 0.285645000 |
| N | -3.242208000 | -0.719195000 | -0.961725000 |
| O | -3.145847000 | -0.282722000 | -2.265528000 |
| C | -3.109771000 | -2.359362000 | 0.637178000 |
| H | -5.670243000 | -1.718843000 | 0.062632000 |
| H | -8.165836000 | -1.852228000 | 0.068561000 |
| C | -3.075462000 | -2.030575000 | -0.763642000 |
| H | -2.049667000 | -3.770612000 | 1.867835000 |
| H | -3.803104000 | -3.966527000 | 1.874672000 |
| C | -2.946135000 | -3.719428000 | 1.230008000 |
| H | -1.758057000 | -2.889488000 | -2.230277000 |
| C | -2.800729000 | -2.980249000 | -1.881872000 |
| H | -2.859286000 | -4.485880000 | 0.452288000 |
| H | -3.446470000 | -2.756329000 | -2.741872000 |
| H | -2.966654000 | -4.018785000 | -1.573713000 |
| H | 6.813574000 | -1.600069000 | 2.443707000 |
| C | 5.299677000 | -0.233146000 | 1.969271000 |
| C | 6.463133000 | -0.601873000 | 2.743472000 |
| N | 4.346545000 | 0.051083000 | 1.364235000 |
| H | 6.200258000 | -0.625471000 | 3.811114000 |
| H | 7.274578000 | 0.122749000 | 2.588716000 |

**CH3Co(DmgBF2)2Acn**

| | | | |
|---|---|---|---|
| Co | 0.464277000 | -0.129408000 | 8.071807000 |
| N | 0.204977000 | -1.995651000 | 8.032632000 |
| N | -1.350807000 | 0.372018000 | 7.990027000 |
| N | 2.279013000 | -0.643910000 | 7.988201000 |
| N | 0.714065000 | 1.737280000 | 7.943244000 |
| C | -1.595220000 | 1.656870000 | 7.896219000 |
| C | -0.374476000 | 2.464267000 | 7.864010000 |
| C | 1.288317000 | -2.732106000 | 7.977506000 |
| C | 2.514718000 | -1.933206000 | 7.946314000 |
| O | 3.308568000 | 0.220638000 | 7.846746000 |
| O | 1.918520000 | 2.334732000 | 7.802054000 |
| O | -0.997626000 | -2.604519000 | 7.933570000 |
| O | -2.386050000 | -0.491982000 | 7.897880000 |
| C | -2.964788000 | 2.226429000 | 7.771667000 |
| C | -0.384555000 | 3.945675000 | 7.717519000 |
| C | 1.270058000 | -4.218155000 | 7.893613000 |
| C | 3.872006000 | -2.536312000 | 7.843349000 |
| B | 3.097418000 | 1.608397000 | 8.408963000 |
| B | -2.173581000 | -1.854858000 | 8.516996000 |
| H | 2.170896000 | -4.648116000 | 8.347316000 |
| H | 1.235517000 | -4.543081000 | 6.841008000 |
| H | 0.379256000 | -4.615373000 | 8.394430000 |
| H | 4.058659000 | -3.210267000 | 8.692794000 |
| H | 3.959085000 | -3.135808000 | 6.924668000 |
| H | 4.637721000 | -1.754816000 | 7.832888000 |
| H | -3.690739000 | 1.585297000 | 8.285643000 |
| H | -3.007923000 | 3.239994000 | 8.187333000 |
| H | -3.260925000 | 2.284527000 | 6.711616000 |
| H | -0.824594000 | 4.234593000 | 6.750702000 |
| H | 0.633888000 | 4.341716000 | 7.775242000 |
| H | -0.996498000 | 4.406700000 | 8.506465000 |
| F | -2.033921000 | -1.739677000 | 9.904902000 |
| F | -3.292042000 | -2.596114000 | 8.150989000 |
| F | 2.968080000 | 1.552878000 | 9.801574000 |

| | | | |
|---|---|---|---|
| F | 4.213205000 | 2.333157000 | 8.003141000 |
| C | 0.514022000 | -0.132523000 | 6.067015000 |
| H | 0.577377000 | -1.169283000 | 5.717284000 |
| H | -0.397498000 | 0.345612000 | 5.690547000 |
| H | 1.402039000 | 0.433864000 | 5.762583000 |
| N | 0.472572000 | -0.089321000 | 10.032524000 |
| C | 0.491054000 | -0.057831000 | 11.190063000 |
| C | 0.516445000 | -0.015942000 | 12.632715000 |
| H | 1.107558000 | 0.846486000 | 12.970723000 |
| H | 0.969568000 | -0.936764000 | 13.025238000 |
| H | -0.506804000 | 0.073521000 | 13.022779000 |

**CH3Ni(Triphos)Co(dmgBF2)2Py**

| | | | |
|---|---|---|---|
| Ni | 2.118638000 | 0.175579000 | 0.248839000 |
| P | 1.614608000 | -1.903642000 | -0.068930000 |
| P | 1.191944000 | -0.040846000 | 2.229105000 |
| P | 1.637407000 | 2.284129000 | 0.442610000 |
| C | 0.290971000 | -1.673767000 | -2.492300000 |
| H | -0.146810000 | -0.774874000 | -2.052992000 |
| C | -0.071141000 | -2.052757000 | -3.784674000 |
| H | -0.794449000 | -1.450627000 | -4.336785000 |
| C | 0.486790000 | -3.199731000 | -4.360805000 |
| H | 0.207176000 | -3.492952000 | -5.374238000 |
| C | 1.402585000 | -3.969565000 | -3.637453000 |
| H | 1.839633000 | -4.864538000 | -4.084008000 |
| C | 1.761371000 | -3.599761000 | -2.337392000 |
| H | 2.472810000 | -4.205774000 | -1.774158000 |
| C | 1.203017000 | -2.451407000 | -1.757788000 |
| C | 4.175595000 | -2.816576000 | 0.467416000 |
| H | 4.483321000 | -1.889280000 | -0.021457000 |
| C | 5.134751000 | -3.692533000 | 0.978174000 |
| H | 6.194945000 | -3.452945000 | 0.881077000 |
| C | 4.734921000 | -4.866470000 | 1.625445000 |
| H | 5.483790000 | -5.546302000 | 2.035296000 |
| C | 3.375182000 | -5.167579000 | 1.750541000 |
| H | 3.060816000 | -6.081825000 | 2.256811000 |
| C | 2.412120000 | -4.299144000 | 1.229940000 |
| H | 1.354966000 | -4.549867000 | 1.330275000 |
| C | 2.807107000 | -3.116655000 | 0.585498000 |
| C | 0.033550000 | -2.146155000 | 0.857844000 |
| H | -0.715275000 | -1.574145000 | 0.290126000 |
| H | -0.281153000 | -3.199224000 | 0.840217000 |
| C | 0.162739000 | -1.592986000 | 2.277728000 |
| H | 0.695205000 | -2.298772000 | 2.930572000 |
| H | -0.820142000 | -1.387184000 | 2.725421000 |
| C | 3.617355000 | -0.615725000 | 3.463225000 |
| H | 3.959451000 | -0.841538000 | 2.451010000 |
| C | 4.490434000 | -0.758091000 | 4.544023000 |
| H | 5.514601000 | -1.094098000 | 4.372462000 |
| C | 4.053730000 | -0.462712000 | 5.839044000 |
| H | 4.736787000 | -0.568639000 | 6.683612000 |
| C | 2.740141000 | -0.030729000 | 6.052722000 |
| H | 2.396358000 | 0.198096000 | 7.062984000 |
| C | 1.862610000 | 0.109834000 | 4.975033000 |
| H | 0.838789000 | 0.443532000 | 5.154940000 |
| C | 2.298959000 | -0.180847000 | 3.672137000 |
| C | 0.107054000 | 1.429010000 | 2.571620000 |
| H | -0.882043000 | 1.113829000 | 2.931953000 |
| H | 0.594083000 | 2.008269000 | 3.368786000 |
| C | -0.004683000 | 2.265569000 | 1.293445000 |
| H | -0.347532000 | 3.289261000 | 1.497933000 |
| H | -0.719631000 | 1.802728000 | 0.596980000 |
| C | 2.282165000 | 4.559505000 | 1.986682000 |
| H | 1.329992000 | 4.980476000 | 1.657270000 |
| C | 3.090079000 | 5.282162000 | 2.865455000 |
| H | 2.763383000 | 6.260603000 | 3.221830000 |
| C | 4.313963000 | 4.752115000 | 3.291899000 |
| H | 4.940993000 | 5.318639000 | 3.982561000 |
| C | 4.732352000 | 3.499500000 | 2.835065000 |
| H | 5.685012000 | 3.083342000 | 3.167003000 |
| C | 3.925500000 | 2.773272000 | 1.954967000 |
| H | 4.243636000 | 1.790636000 | 1.600901000 |
| C | 2.696529000 | 3.297398000 | 1.527004000 |

| | | | |
|---|---|---|---|
| C | 2.252431000 | 4.288681000 | -1.446406000 |
| H | 3.084558000 | 4.561286000 | -0.795434000 |
| C | 2.060387000 | 4.968447000 | -2.652764000 |
| H | 2.748302000 | 5.766563000 | -2.937605000 |
| C | 0.993292000 | 4.629158000 | -3.489679000 |
| H | 0.843812000 | 5.163482000 | -4.429496000 |
| C | 0.117589000 | 3.602391000 | -3.119812000 |
| H | -0.719698000 | 3.330192000 | -3.764776000 |
| C | 0.309209000 | 2.914464000 | -1.921158000 |
| H | -0.383045000 | 2.112558000 | -1.657630000 |
| C | 1.370513000 | 3.264936000 | -1.067513000 |
| C | 2.919326000 | 0.397931000 | -1.561354000 |
| H | 3.412015000 | -0.520460000 | -1.911314000 |
| H | 3.647103000 | 1.223498000 | -1.523506000 |
| H | 2.119448000 | 0.662434000 | -2.267918000 |
| H | -3.185710000 | 4.890802000 | 2.083890000 |
| H | -2.205661000 | 3.825916000 | 3.117050000 |
| H | -3.965324000 | 3.807125000 | 3.271086000 |
| H | -3.232260000 | 5.136809000 | 0.002978000 |
| C | -3.145010000 | 3.897611000 | 2.544658000 |
| C | -2.893159000 | 4.293156000 | -0.609430000 |
| H | -1.827386000 | 4.448622000 | -0.844010000 |
| H | -3.435901000 | 4.303245000 | -1.564113000 |
| C | -3.234442000 | 2.819828000 | 1.516531000 |
| C | -3.113028000 | 2.994917000 | 0.092973000 |
| O | -3.400736000 | 1.250822000 | 3.192561000 |
| N | -3.379710000 | 1.532377000 | 1.842884000 |
| H | -5.669309000 | 2.404321000 | 0.521016000 |
| H | -8.156044000 | 2.532489000 | 0.315932000 |
| F | -3.731212000 | -0.225878000 | 4.931387000 |
| B | -4.017334000 | -0.060544000 | 3.564784000 |
| N | -3.164341000 | 1.823151000 | -0.550040000 |
| F | -5.414121000 | -0.027792000 | 3.345576000 |
| C | -6.268667000 | 1.505573000 | 0.358309000 |
| C | -7.654110000 | 1.567339000 | 0.242716000 |
| O | -2.958908000 | 1.865177000 | -1.912096000 |
| Co | -3.586134000 | 0.313922000 | 0.454996000 |
| N | -5.589664000 | 0.343593000 | 0.276807000 |
| O | -3.411944000 | -1.250920000 | 2.891220000 |
| C | -8.372554000 | 0.387530000 | 0.034658000 |
| H | -9.459036000 | 0.404685000 | -0.059763000 |
| N | -3.390343000 | -1.201443000 | 1.513021000 |
| B | -3.484714000 | 0.694245000 | -2.679043000 |
| F | -4.899667000 | 0.716852000 | -2.701104000 |
| F | -2.966011000 | 0.844759000 | -3.978839000 |
| C | -6.280494000 | -0.796787000 | 0.075795000 |
| C | -7.666325000 | -0.814756000 | -0.049712000 |
| N | -3.183363000 | -0.916590000 | -0.881744000 |
| O | -2.990692000 | -0.638249000 | -2.215886000 |
| C | -3.265804000 | -2.376220000 | 0.888646000 |
| H | -5.690471000 | -1.714254000 | 0.015645000 |
| H | -8.178071000 | -1.763825000 | -0.211582000 |
| C | -3.145009000 | -2.208410000 | -0.536101000 |
| H | -2.311057000 | -3.727670000 | 2.265209000 |
| H | -4.075889000 | -3.766077000 | 2.303599000 |
| C | -3.210355000 | -3.669302000 | 1.631663000 |
| H | -1.892638000 | -3.358649000 | -1.856325000 |
| C | -2.944241000 | -3.301361000 | -1.532316000 |
| H | -3.198938000 | -4.522549000 | 0.944863000 |
| H | -3.544388000 | -3.109019000 | -2.432353000 |
| H | -3.228167000 | -4.274788000 | -1.116468000 |

**Ni(Triphos)**

| | | | |
|---|---|---|---|
| Ni | 2.269717000 | 0.150444000 | 0.918907000 |
| P | 1.713240000 | -1.765682000 | 0.174859000 |
| P | 1.385923000 | -0.135705000 | 2.841488000 |
| P | 1.218404000 | 1.963855000 | 0.546165000 |
| C | 0.691509000 | -1.003496000 | -2.310615000 |
| H | 0.887708000 | 0.003257000 | -1.933362000 |
| C | 0.119894000 | -1.190271000 | -3.572728000 |
| H | -0.139004000 | -0.322552000 | -4.183673000 |
| C | -0.106577000 | -2.484730000 | -4.052324000 |
| H | -0.542612000 | -2.634873000 | -5.041939000 |

| | | | |
|---|---|---|---|
| C | 0.233714000 | -3.589172000 | -3.261682000 |
| H | 0.061195000 | -4.601211000 | -3.634318000 |
| C | 0.793252000 | -3.401083000 | -1.994583000 |
| H | 1.056960000 | -4.266881000 | -1.382898000 |
| C | 1.026278000 | -2.104233000 | -1.507455000 |
| C | 3.984118000 | -3.297516000 | -0.321143000 |
| H | 4.200628000 | -2.512897000 | -1.051493000 |
| C | 4.888326000 | -4.344056000 | -0.140235000 |
| H | 5.804236000 | -4.378722000 | -0.733810000 |
| C | 4.627048000 | -5.343084000 | 0.806776000 |
| H | 5.338059000 | -6.157597000 | 0.956334000 |
| C | 3.450901000 | -5.288531000 | 1.559108000 |
| H | 3.237408000 | -6.063637000 | 2.298169000 |
| C | 2.537048000 | -4.245949000 | 1.366952000 |
| H | 1.619930000 | -4.231847000 | 1.957682000 |
| C | 2.790353000 | -3.236895000 | 0.423719000 |
| C | 0.190565000 | -2.114595000 | 1.206105000 |
| H | -0.597266000 | -1.508217000 | 0.734135000 |
| H | -0.117604000 | -3.167871000 | 1.107260000 |
| C | 0.379687000 | -1.711590000 | 2.673377000 |
| H | 0.951369000 | -2.480348000 | 3.213481000 |
| H | -0.587525000 | -1.603282000 | 3.189657000 |
| C | 2.974661000 | -1.352579000 | 4.766159000 |
| H | 3.273364000 | -1.990077000 | 3.928093000 |
| C | 3.552626000 | -1.546922000 | 6.020232000 |
| H | 4.286349000 | -2.343314000 | 6.161596000 |
| C | 3.204573000 | -0.713425000 | 7.091647000 |
| H | 3.664114000 | -0.856180000 | 8.071307000 |
| C | 2.267912000 | 0.304791000 | 6.894171000 |
| H | 1.988394000 | 0.958535000 | 7.723242000 |
| C | 1.674655000 | 0.489404000 | 5.640037000 |
| H | 0.934873000 | 1.281485000 | 5.517771000 |
| C | 2.016577000 | -0.339272000 | 4.556968000 |
| C | 0.117391000 | 1.221347000 | 2.999743000 |
| H | -0.753015000 | 0.902812000 | 3.594755000 |
| H | 0.608932000 | 2.046711000 | 3.538616000 |
| C | -0.303534000 | 1.710485000 | 1.608839000 |
| H | -0.890352000 | 2.640000000 | 1.675270000 |
| H | -0.923100000 | 0.952328000 | 1.105965000 |
| C | 1.259906000 | 4.810437000 | 0.891983000 |
| H | 0.429567000 | 4.853359000 | 0.183642000 |
| C | 1.743845000 | 5.987700000 | 1.468797000 |
| H | 1.287716000 | 6.945978000 | 1.211250000 |
| C | 2.809764000 | 5.940813000 | 2.375179000 |
| H | 3.186658000 | 6.861781000 | 2.824143000 |
| C | 3.392681000 | 4.711591000 | 2.700211000 |
| H | 4.227087000 | 4.669493000 | 3.403298000 |
| C | 2.912495000 | 3.535175000 | 2.117169000 |
| H | 3.361861000 | 2.567512000 | 2.360370000 |
| C | 1.839594000 | 3.571598000 | 1.211786000 |
| C | 1.424613000 | 2.827565000 | -2.088256000 |
| H | 2.495153000 | 2.842564000 | -1.864801000 |
| C | 0.982551000 | 3.125848000 | -3.376302000 |
| H | 1.705177000 | 3.391872000 | -4.150455000 |
| C | -0.384368000 | 3.066691000 | -3.681228000 |
| H | -0.731103000 | 3.283426000 | -4.693217000 |
| C | -1.298386000 | 2.725616000 | -2.681959000 |
| H | -2.365443000 | 2.678673000 | -2.909437000 |
| C | -0.855797000 | 2.445007000 | -1.383530000 |
| H | -1.589725000 | 2.187712000 | -0.619235000 |
| C | 0.512712000 | 2.487143000 | -1.070285000 |

**CH3Ni(triphos)Acn**

| | | | |
|---|---|---|---|
| Ni | 2.205153000 | -0.072968000 | 0.528223000 |
| P | 1.126348000 | -1.949148000 | 0.220619000 |
| P | 1.219267000 | -0.012551000 | 2.505226000 |
| P | 1.284314000 | 1.896378000 | 0.194506000 |
| C | -0.942535000 | -1.897648000 | -1.702182000 |
| H | -1.572818000 | -1.444781000 | -0.935509000 |
| C | -1.462018000 | -2.076387000 | -2.987367000 |
| H | -2.487887000 | -1.769721000 | -3.201001000 |
| C | -0.673190000 | -2.643650000 | -3.992472000 |

| | | | |
|---|---|---|---|
| H | -1.079489000 | -2.782202000 | -4.996221000 |
| C | 0.638513000 | -3.036282000 | -3.704658000 |
| H | 1.261016000 | -3.480809000 | -4.483817000 |
| C | 1.159334000 | -2.861429000 | -2.421212000 |
| H | 2.182180000 | -3.176067000 | -2.206266000 |
| C | 0.371751000 | -2.293904000 | -1.403570000 |
| C | 3.301885000 | -3.416873000 | 1.190057000 |
| H | 3.741486000 | -2.434706000 | 1.372278000 |
| C | 3.990972000 | -4.581688000 | 1.544281000 |
| H | 4.977721000 | -4.509809000 | 2.006284000 |
| C | 3.417281000 | -5.832891000 | 1.303530000 |
| H | 3.954798000 | -6.743228000 | 1.576686000 |
| C | 2.151544000 | -5.919900000 | 0.709354000 |
| H | 1.701445000 | -6.896794000 | 0.520909000 |
| C | 1.462078000 | -4.758801000 | 0.356943000 |
| H | 0.476708000 | -4.832187000 | -0.108488000 |
| C | 2.036107000 | -3.498101000 | 0.594917000 |
| C | -0.279582000 | -2.026029000 | 1.431396000 |
| H | -1.069549000 | -1.346229000 | 1.080130000 |
| H | -0.694335000 | -3.043307000 | 1.474660000 |
| C | 0.263896000 | -1.568994000 | 2.784808000 |
| H | 0.953834000 | -2.324842000 | 3.186706000 |
| H | -0.533073000 | -1.401572000 | 3.523770000 |
| C | 2.838475000 | 1.521074000 | 4.168401000 |
| H | 2.549332000 | 2.360347000 | 3.532812000 |
| C | 3.768313000 | 1.731704000 | 5.187095000 |
| H | 4.178741000 | 2.731236000 | 5.343694000 |
| C | 4.180208000 | 0.665796000 | 5.994639000 |
| H | 4.909684000 | 0.830286000 | 6.789904000 |
| C | 3.656322000 | -0.611229000 | 5.775420000 |
| H | 3.974702000 | -1.449269000 | 6.398529000 |
| C | 2.724483000 | -0.826417000 | 4.755670000 |
| H | 2.338408000 | -1.833898000 | 4.597811000 |
| C | 2.295076000 | 0.241365000 | 3.951560000 |
| C | -0.032968000 | 1.365429000 | 2.559178000 |
| H | -0.947174000 | 0.996994000 | 3.046797000 |
| H | 0.382094000 | 2.159573000 | 3.194732000 |
| C | -0.304396000 | 1.886814000 | 1.147019000 |
| H | -0.785468000 | 2.875511000 | 1.155184000 |
| H | -0.964903000 | 1.202347000 | 0.594108000 |
| C | 1.651119000 | 4.343097000 | 1.615355000 |
| H | 0.574375000 | 4.363052000 | 1.790411000 |
| C | 2.447925000 | 5.355963000 | 2.157502000 |
| H | 1.985669000 | 6.146828000 | 2.751705000 |
| C | 3.829063000 | 5.357720000 | 1.939486000 |
| H | 4.449037000 | 6.148493000 | 2.366439000 |
| C | 4.413967000 | 4.343235000 | 1.173391000 |
| H | 5.491550000 | 4.340182000 | 0.997117000 |
| C | 3.622194000 | 3.322263000 | 0.645364000 |
| H | 4.083766000 | 2.518048000 | 0.070305000 |
| C | 2.232903000 | 3.312696000 | 0.859750000 |
| C | 1.163503000 | 3.662321000 | -2.031741000 |
| H | 1.782823000 | 4.353073000 | -1.457325000 |
| C | 0.753191000 | 4.007377000 | -3.323831000 |
| H | 1.059161000 | 4.965475000 | -3.749143000 |
| C | -0.047072000 | 3.134292000 | -4.066865000 |
| H | -0.366119000 | 3.407360000 | -5.074643000 |
| C | -0.433548000 | 1.906325000 | -3.516362000 |
| H | -1.052597000 | 1.214539000 | -4.091719000 |
| C | -0.016829000 | 1.555309000 | -2.232130000 |
| H | -0.300357000 | 0.586002000 | -1.816860000 |
| C | 0.775584000 | 2.435021000 | -1.474581000 |
| C | 2.908085000 | -0.160790000 | -1.350905000 |
| H | 3.496318000 | -1.084670000 | -1.443648000 |
| H | 3.560686000 | 0.711065000 | -1.495742000 |
| H | 2.099456000 | -0.153291000 | -2.089965000 |
| N | 4.013319000 | 0.027548000 | 1.243605000 |
| C | 5.031739000 | 0.217183000 | 1.775465000 |
| C | 6.280261000 | 0.471578000 | 2.456688000 |
| H | 6.633429000 | 1.487314000 | 2.225009000 |
| H | 6.131043000 | 0.386290000 | 3.543199000 |
| H | 7.044226000 | -0.253494000 | 2.141393000 |

Table S1.. Total energies of molecules considered in the calculations

| | BP86 | BP86-D3Bj | PBE | PBE-D3BJ | PBE-D3BJ | PBE-D3BJ | PBE-D4 | PBE-SCNL |
|---|---|---|---|---|---|---|---|---|
| | | | | | | | | |
| | ENERGY | ENERGY | ENERGY | ENERGY | GIBBS ENERGY | GIBBS ENER-SCAL | ENERGY | ENERGY |
| acn | -132,8078107 | -132,8119348 | -132,6387351 | -132,641543 | -132,621598 | -132,6078715 | -132,6443 | -132,5597166 |
| **codmg-acn** | -2797,866398 | -2797,952152 | -2795,850641 | -2795,903416 | -2795,689638 | -2795,670827 | -2795,91959 | -2795,114146 |
| **codmg-acn-r** | -2797,701109 | -2797,79698 | -2795,700279 | -2795,753434 | -2795,689638 | | -2795,76747 | -2794,963897 |
| **codmg** | -2665,043769 | -2665,115229 | -2663,194231 | -2663,238207 | -2663,063689 | -2663,045075 | -2663,2508 | -2662,527696 |
| **codmg-met-acn** | -2837,626714 | -2837,726506 | -2835,556509 | -2835,618231 | -2835,367075 | -2835,348234 | -2835,63477 | -2834,799153 |
| **codmg-met-acn-m** | -2837,84854 | -2837,846858 | -2835,742719 | -2835,745868 | | | -2835,75188 | -2834,914384 |
| **codmg-met** | -2704,797632 | -2704,882271 | -2702,893056 | -2702,945279 | -2702,73138 | -2702,712706 | -2951,07211 | -2950,16329 |
| **codmg-met-m** | -2704,95122 | -2705,035207 | -2703,042443 | -2703,094088 | | | -2951,18947 | -2950,277862 |
| **codmgpy-** | -2913,433665 | -2913,541151 | -2911,273759 | -2911,339836 | -2911,082064 | -2911,063105 | -2911,35722 | -2910,478473 |
| **codmgpy-met** | -2953,192993 | -2953,315397 | -2950,978602 | -2951,054263 | -2950,760066 | -2950,741064 | -2951,0721 | -2950,163274 |
| **codmgpy-met-m** | -2953,315865 | -2953,43605 | -2951,096881 | -2951,171167 | -2950,881761 | -2950,862759 | -2951,18947 | -2950,277861 |
| **codmgpy-r** | -2913,279364 | -2913,38739 | -2911,124029 | -2911,190431 | -2910,933619 | -2910,914661 | -2911,20585 | -2910,328975 |
| **codmg-r** | -2664,88662 | -2664,958314 | | -2663,086146 | | | -2663,09638 | -2662,375795 |
| **codmg-t** | | | | | | | | |
| **nip3-0** | -3848,76939 | -3848,984919 | -3846,018213 | -3846,14546 | -3845,664212 | -3845,644514 | -3846,15891 | -3844,98609 |
| **nip3+** | -3848,632893 | -3848,848085 | -3845,885591 | -3846,013054 | -3845,528584 | -3845,508903 | -3846,02592 | -3844,854511 |
| **nip3-met-acn** | -4021,373482 | -4021,629429 | -4018,402225 | -4018,554394 | -4017,993995 | -4017,97421 | -4018,57272 | -4017,286332 |
| **nip3-met** | -3888,560248 | -3888,795397 | -3885,75407 | -3885,892082 | -3885,373481 | -3885,353797 | -3885,90945 | -3884,704301 |
| **nip4** | -4885,355445 | -4885,719689 | -4881,529208 | -4881,746253 | -4881,015856 | -4880,995586 | -4881,76891 | -4880,074307 |
| **nip4-p** | -4885,206526 | -4885,57741 | -4881,38456 | -4881,603381 | -4880,871139 | -4880,850869 | -4881,62655 | -4879,93363 |
| **PPh3** | -1036,555136 | -1036,636485 | -1035,469844 | -1035,519604 | -1035,301409 | -1035,283275 | -1035,52726 | -1035,00602 |
| **Met** | -39,8509738 | -39,85210465 | -39,78801963 | -39,78886796 | -39,779709 | -39,76828475 | -39,7893726 | -39,75440168 |
| **Py** | -248,3666269 | -248,3819298 | -248,0521125 | -248,0620265 | -248,003394 | -247,9880532 | -248,064567 | -247,9068591 |

**Table S2.** Reaction energies in kcal/mol. Entries *b* and *d* are redox potentials in Volts..

| Reaction | BP86 ΔE | BP86-D3BJ ΔE | PBE ΔE | PBE-D3BJ ΔE | PBE-D3BJ ΔG | PBE-D4 ΔE | PBE-SCNL ΔE |
|---|---|---|---|---|---|---|---|
| Total reaction energy | | | | | | | |
| 4 | 0.4 | -38.9 | -6.4 | -30.7 | -22.88 | -29.5 | -30.6 |
| 5 | – | -15.2 | – | -12.2 | -7.08 | -13.0 | -12.3 |
| 6 | 3.8 | -25.1 | -0.4 | -17.7 | -15.01 | -17.6 | -16.6 |
| 7 | – | -1.1 | – | 1.1 | 1.85 | -0.3 | 2.9 |
| 18 | -1.5 | -37.0 | -6.6 | -27.7 | -22.94 | -28.5 | -27.3 |
| 19 | – | -13.1 | – | -8.9 | -6.08 | -11.1 | -7.8 |
| Elementary reactions energy | | | | | | | |
| 8 | 19.4 | 61.6 | 25.8 | 50.9 | 42.54 | 51.9 | 51.6 |
| 9 | 11.6 | 58.2 | 18.3 | 44.4 | 36.82 | 46.0 | 45.7 |
| 10 | 19.6 | 26.8 | 22.0 | 26.6 | 19.68 | 26.9 | 28.2 |
| 11 | 39.3 | 47.6 | 41.8 | 47.0 | 36.46 | 48.2 | 50.1 |
| 12 | 47.9 | 59.7 | 50.5 | 56.6 | 48.07 | 59.0 | 59.8 |
| 13 | 3.4 | 13.8 | 5.9 | 13.0 | 7.86 | 11.9 | 14.0 |
| 14 | 5.3 | 11.9 | 6.1 | 10.0 | 7.93 | 10.8 | 10.7 |
| 15[a)] | -0.3 | -0.48 | -0.4 | -0.5 | -0.42 | -0.4 | -0.5 |
| 16[b)] | -1.0 | -1.07 | -1.1 | -1.2 | -1.04 | -1.1 | -1.2 |

a) Expt -0.1 V (Ref.[31]). b) Expt -1.1 V (Ref.[31]).
